# The *Staphylococcus aureus* esterase FmtA is essential for wall teichoic acid D-alanylation

**DOI:** 10.1128/mbio.02337-25

**Published:** 2025-09-12

**Authors:** Kirsten A. Berry, Mackenzie T. A. Verhoef, Zhiyong Zheng, Ronald S. Flannagan, Telmo O. Paiva, Stephanie E. Gilbert, M. Sameer Al-Abdul-Wahid, David E. Heinrichs, Yves F. Dufrêne, Georgina Cox

**Affiliations:** 1 Department of Molecular and Cellular Biology, University of Guelph 317113, Guelph, Ontario, Canada; 2 Louvain Institute of Biomolecular Science and Technology, UCLouvain 195141, Louvain-la-Neuve, Belgium; 3 Department of Microbiology and Immunology, University of Western Ontario 6221, London, Ontario, Canada; 4 Advanced Analysis Centre, University of Guelph 3653, Guelph, Ontario, Canada; Georgia Institute of Technology, Atlanta, Georgia, USA; University of Pittsburgh, Pittsburgh, Pennsylvania, USA

**Keywords:** *Staphylococcus aureus*, methicillin-resistant *Staphylococcus aureus*, teichoic acid, wall teichoic acid, virulence, bacterial host adhesion, antibiotic resistance, cell surface charge

## Abstract

*Staphylococcus aureus* teichoic acids are anionic glycopolymers covalently attached to peptidoglycan (wall teichoic acids, WTAs) or anchored to the phospholipid membrane (lipoteichoic acids, LTAs). The post-synthetic addition of D-alanine (D-Ala) residues to these polymers modulates surface charge and contributes to pathogen survival in the host environment. Despite this importance, the underlying mechanisms controlling WTA D-alanylation remain a significant area for further investigation. Here, we demonstrate that the teichoic acid D-Ala esterase, FmtA, is essential for WTA D-alanylation. The inactivation of *fmtA* results in a more negative net surface charge, impacting host adhesion, biofilm formation, and cell aggregation. We found that LTA from *fmtA*-deficient strains retains normal D-alanylation levels, while WTA is almost devoid of D-Ala. These data support the notion that LTA provides the D-Ala for WTA modification, a process dependent on FmtA.

**IMPORTANCE:**

The D-alanine (D-Ala) modification of *Staphylococcus aureus* teichoic acids influences bacterial interactions and survival under stress. While this modification is important for host survival, the mechanisms underlying wall teichoic acid (WTA) D-alanylation remain unclear. A deeper understanding of this process could lead to the development of targeted therapies to combat *S. aureus* infections. We have identified FmtA as essential for this process, supporting the idea that lipoteichoic acid (LTA) provides the D-Ala used to modify WTAs. Our findings highlight a critical gap in understanding this mechanism: an acyltransferase must incorporate the D-Ala released from LTAs by FmtA into WTAs.

## INTRODUCTION

A bacterium’s ability to colonize and infect a host critically depends on its cell envelope. This structure protects against the environment, mediates interactions with host cells, and directly influences resistance to immune responses and disease severity (1). In the critical pathogen *Staphylococcus aureus*, the cell envelope consists of a cytoplasmic membrane, the peptidoglycan, teichoic acids (TAs), and a variety of proteins (1, 2).

The TAs within the *S. aureus* cell envelope are particularly abundant, accounting for up to 60% of the cell wall mass (3–5). These anionic glycopolymers are either covalently attached to the peptidoglycan (wall teichoic acids, WTAs) or anchored to cell membrane glycolipids (lipoteichoic acids, LTAs) (4, 6). LTAs and WTAs are biosynthetically distinct and differ in the composition of their repeating subunits. Notably, while LTAs remain localized within the cell wall (7), WTAs project beyond it, extending outward from the bacterial surface (3, 8, 9). In *S. aureus*, LTAs are composed of polyglycerol-phosphate (Gro-P), polymerized outside of the cell by the LTA synthase, LtaS (6, 7, 10, 11). In contrast, WTAs contain a polyribitol-phosphate (Rbo-P) backbone, which is synthesized intracellularly on an undecaprenyl phosphate carrier (6, 10). Once polymerized, WTAs are transported across the cytoplasmic membrane by the TarGH ABC system and then transferred from the undecaprenol lipid carrier to muramic acid residues within the peptidoglycan (10, 12). Importantly, both of these polymers are post-synthetically glycosylated with *N*-acetylglucosamine (GlcNAc) and esterified with D-alanine (D-Ala) residues (13–15).

The anionic phosphate groups within the TA backbone are responsible for the moderately negative net charge on the *S. aureus* cell surface at neutral pH, and modification of this backbone through D-Ala substitution influences surface charge (16). TA D-Ala esterification occurs in the extracellular environment, which poses a challenge due to the lack of an external energy source (13, 17, 18). For the LTAs, this process is facilitated by the D-alanyl-lipoteichoic acid (Dlt) pathway, encoded by the *dlt* operon, comprising a series of proteins and enzymes (DltA, DltB, DltC, DltD, and DltX) (13, 19, 20). The *dlt* operon is upregulated by the GraRS two-component system (TCS), leading to increased D-alanylation of TAs (21). DltA functions to activate D-Ala, producing the high-energy intermediate D-alanyl-AMP (13, 18). The D-alanyl group is subsequently transferred to the carrier protein DltC, forming a D-alanyl thioester (13, 17, 18, 22, 23). DltC interacts with DltB, a membrane-bound O-acetyltransferase, facilitating the transport of D-alanyl across the membrane (13, 18, 20). DltX forms a complex with DltB and DltD (13); it is proposed that D-Ala is shuttled across this protein complex before being covalently attached to the C2 hydroxyl group of LTA (13).

Despite considerable research on *S. aureus* cell wall structure, the enzymatic mechanism of WTA D-alanylation remains elusive. Current models propose that D-Ala esters associated with LTA serve as donors for this modification, but the underlying process is poorly defined (20, 24). Nonetheless, the formyl methionyl transferase A (FmtA) enzyme in *S. aureus* has been associated with WTA D-alanylation; while the protein was initially identified for its role in methicillin resistance and the cell wall stimulon (25, 26), it has since been shown to localize to the division septum (27) where it hydrolyzes the ester bond between D-Ala residues decorating TAs (28, 29). FmtA may remove D-Ala from LTAs, making them available for incorporation into WTAs (29); however, this has not been demonstrated, and the enzyme responsible for incorporating D-Ala into WTAs is unknown. FmtA was ruled out as a potential candidate due to a lack of transesterase activity (29). Furthermore, FmtA is proposed to remove D-Ala residues from both LTAs and WTAs, potentially allowing the cell to modify its surface properties in response to different environmental conditions (29).

TA D-alanylation is associated with various cellular processes, impacting cell surface charge (30) and influencing diverse functions such as autolytic activity (27, 31), metal chelation (32, 33), and the proper folding of exported proteins (34). This modification is also implicated in biofilm formation (27, 35), adhesion to the inner nasal cavity via interactions with SREC-I (36), resistance to neutrophil killing (37), and overall susceptibility to environmental stressors, including CAMPs (16, 38), antibiotics (31), heat stress (39), and lysozyme (40, 41). Given the importance of these cellular processes, it is therefore somewhat surprising that the underlying mechanisms governing WTA D-alanylation remain poorly defined.

We revealed that disruption of *fmtA* reduced *S. aureus* adhesion to host ligands (42). Current literature suggests that *fmtA* mutants exhibit increased TA D-alanylation due to a deficiency in esterase activity (29), which would presumably result in a more positively charged cell surface. We posited that the adhesion defects measured in an *fmtA* mutant were due to alterations in cell surface charge resulting from impaired esterase activity, which likely impacts electrostatic interactions contributing to the early stages of adhesion (43, 44). In this study, we employed two different approaches to measure the surface charge of *S. aureus*, demonstrating that the loss of *fmtA* resulted in a more negatively charged cell surface, which was unexpected given the anticipated increase in positive charge due to reduced esterase activity. To investigate, we extracted and purified TAs from the *S. aureus* cell envelope and analyzed D-alanylation using ^1^H NMR spectroscopy, revealing that LTA D-alanylation is unchanged in an *fmtA* mutant, but WTA D-alanylation is reduced by ~95%.

The distinct cellular localization of WTA and LTA means that modifications to their respective charges likely exert regionally specific effects. Indeed, we found that changes in WTA-associated surface charge—a property primarily affecting the outermost layer of the *S. aureus* cell surface—influenced host cell adhesion, biofilm formation, and cell aggregation. Conversely, changes in LTA-associated charge, which reside closer to the cell membrane within the peptidoglycan of the cell wall, primarily impacted polymyxin B susceptibility and macrophage replication. In summary, our results indicate that FmtA is essential for WTA D-alanylation, supporting the notion that FmtA releases D-Ala esters from LTA, allowing their incorporation into WTAs. When this process is disrupted, the cell surface becomes more negatively charged, which increases electrostatic repulsion and impacts adhesion and other processes associated with host colonization and infection.

## RESULTS

### *fmtA*-deficient mutants exhibit a more negative net surface charge

To assess the effect of *fmtA* deficiency on *S. aureus* surface charge, we first created a ∆*fmtA* deletion mutant via homologous recombination, as our earlier work employed a transposon mutant (45). To provide a contrasting model with minimal TA D-alanylation, we targeted the *dltXABCD* operon; however, attempts to generate this mutant in the methicillin-resistant *S. aureus* (MRSA) USA300 JE2 clone LAC (JE2) (46) were unsuccessful using both homologous recombination and CRISPR-Cas9-mediated counterselection (47). Instead, we utilized a *graS* transposon mutant (USA300 JE2 *graS*::Tn) (46). As described earlier, the GraRS TCS positively regulates the expression of the *dltXABCD* operon (21, 48). We confirmed a significant reduction in *dltA* expression in JE2 *graS*::Tn using reverse transcription-quantitative polymerase chain reaction (Fig. S1). However, we note that disruption of this TCS has pleiotropic effects, impacting the expression of other determinants of cell surface charge regulation (49), such as *mprF,* which should be considered.

**Fig 1:**
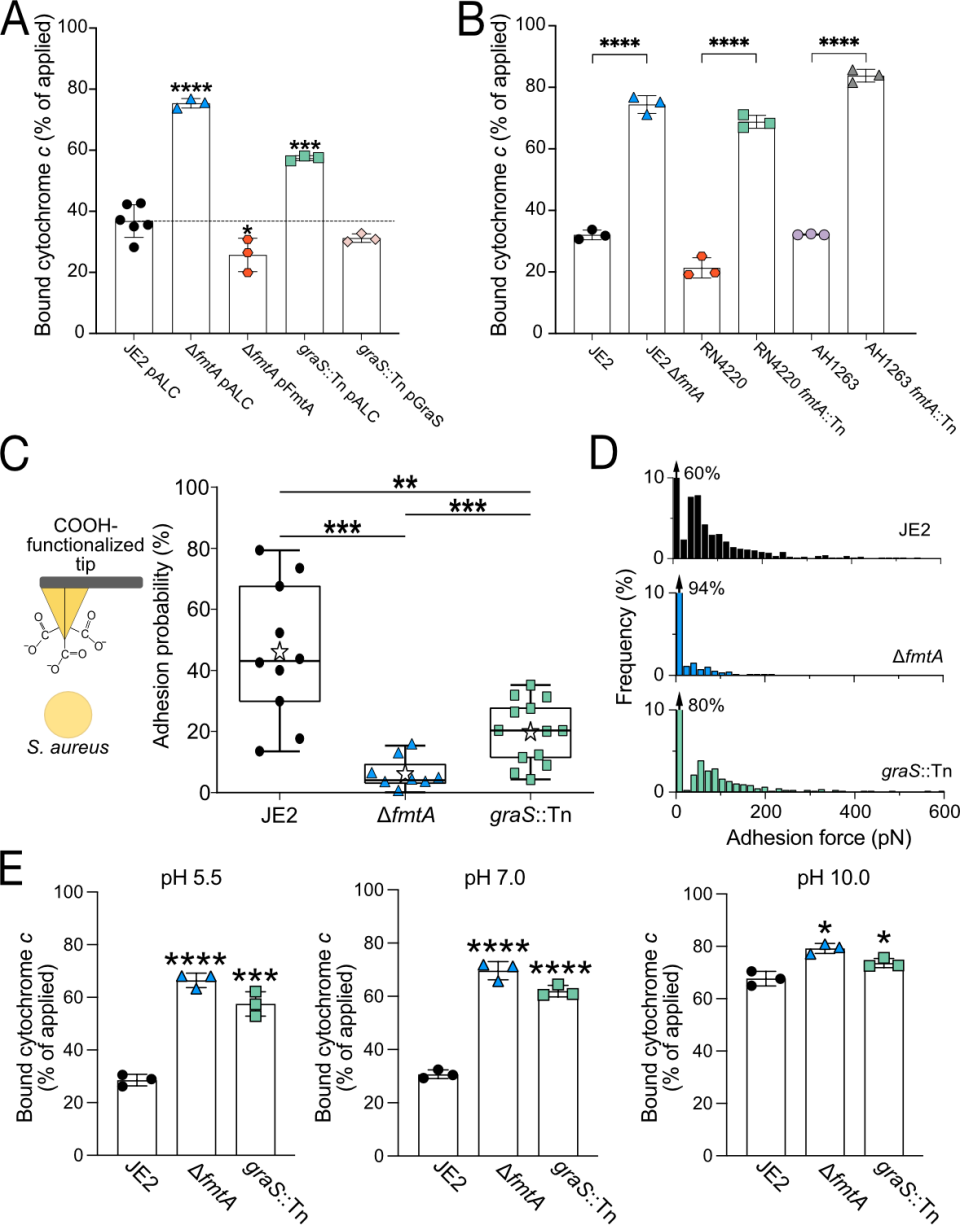
The disruption of *fmtA* and *graS* reduces *S. aureus* surface charge. (**A**) Measuring cytochrome *c* binding (percentage of applied) to MRSA USA300 JE2 (JE2) strains sampled at an OD_600nm_ of 0.5–0.6. Gene complementation using the pALC2073 (pALC) plasmid restored surface charge. An empty pALC vector was used as a control. (**B**) Bound cytochrome *c* (percentage of applied) to different *S. aureus* strains lacking *fmtA*. (**C**) Chemical force microscopy, employing an atomic force microscopy tip functionalized with negatively charged carboxyl-terminated alkanethiols, to measure cell surface charge on single *S. aureus* cells (as depicted in the inset). Each data point represents adhesion probability between the tip and a single *S. aureus* cell. Stars indicate mean values, lines are medians, boxes indicate 25%–75% quartiles, and whiskers indicate standard deviation. (**D**) Representative adhesion force histogram from one cell for each strain. Each histogram represents the adhesion forces calculated from a total of 1,024 force-distance curves spatially probed on the top of one cell. Additional histograms for other cells are presented in Fig. S3. (**E**) Measuring cytochrome *c* binding (percentage of applied) to JE2 grown at various pH values. Related to Fig. S2B. Unless otherwise stated, each data point represents a single biological replicate determined by averaging three technical replicates. Error bars represent the standard deviation of the mean. *P*-values were calculated using the two-tailed unpaired Student’s *t*-test comparing each mutant to the parental strain (JE2). *P*-values are denoted as *P ≤ 0.05, **P ≤ 0.01, ***P ≤ 0.001, and ****P ≤ 0.0001.

To compare the relative net cell surface charge of the ∆*fmtA* and *graS*::Tn mutants at neutral pH, we measured the binding of the small positively charged cytochrome *c* protein to whole cells (50). This analysis showed that significantly more cytochrome *c* molecules interacted with the surfaces of the ∆*fmtA* and *graS*::Tn mutants, providing indirect evidence that the net surface charge of these mutants is more negative compared to the parental strain (Fig. 1A). Gene complementation restored surface charge to or beyond wild-type levels, further validating these findings (Fig. 1A). Cell surface charge was also evaluated at different cell cycle stages, demonstrating the cell surface of ∆*fmtA* was consistently more negative at all stages of the cell cycle compared to the parental strain (Fig. S2A). However, the impact of *graS* disruption diminished in the later stages of the cell cycle (Fig. S2A), likely due to reduced expression of this TCS (51). Disruption of *fmtA* in other *S. aureus* strains (AH1263 and RN4220) demonstrated that the changes in surface charge are not strain specific and are generalizable (Fig. 1B).

We next directly evaluated the surface charge of single cells at neutral pH by chemical force microscopy (52, 53), in which an atomic force microscopy (AFM) tip was functionalized with negatively charged carboxyl-terminated alkanethiols (Fig. 1C) (54).

Adhesion events were probed across an area of 500 × 500 nm^2^ on the top of single live cells (Fig. 1C). The parental strain (JE2) exhibited the highest adhesion probability, followed by JE2-*graS*::Tn, with JE2-Δ*fmtA* cells showing the lowest adhesion probability. This direct approach confirmed that *ΔfmtA* and *graS*::Tn mutants exhibit a more negative cell surface charge than the parental strain. Similar mean adhesion forces were calculated for the three strains (Fig. 1D; Fig. S3): 51 ± 9, 44 ± 13, and 51 ± 13 pN for JE2, JE2-Δ*fmtA*, and JE2-*graS*::Tn, respectively, corroborating that the interactions established between the cell surface and the probe are of the same nature, i.e., electrostatic, but reduced in the mutants.

We hypothesized that the changes in cell surface charge were linked to TA D-Ala content, prompting us to examine the effect of pH. D-Ala is covalently attached to TAs via its C-terminus, and its amino group is ionizable. With a pKa of 9.69, the D-Ala amino group remains positively charged under neutral or acidic conditions but becomes deprotonated in alkaline conditions (above pH 10) (33). Additionally, TA D-Ala residues dissociate under alkaline conditions due to ester bond hydrolysis (55, 56). Thus, at pH 10.0, we expected the surface charges of the parental strain, ∆*fmtA*, and *graS*::Tn mutants to be similar. As anticipated, differences in cytochrome *c* affinity were reduced under alkaline conditions for both the parental strain and the mutants (Fig. 1E; Fig. S2B).

### *fmtA* inactivation impairs wall teichoic acid, but not lipoteichoic acid, D-alanylation

We hypothesized that when FmtA esterase activity (29) was removed due to the deletion of *fmtA*, D-Ala could not be released from LTA for transfer to WTA, thereby rendering the outermost region of the cell surface more negatively charged. Indeed, since WTAs extend beyond the cell surface (3), they likely play a more significant role than LTAs in determining relative net surface charge. To investigate, we extracted and purified TAs from the parental strain (JE2), the ∆*fmtA,* and *graS*::Tn mutants. The TAs were extracted in triplicate for each strain, with three independently purified preparations.

**Fig 2:**
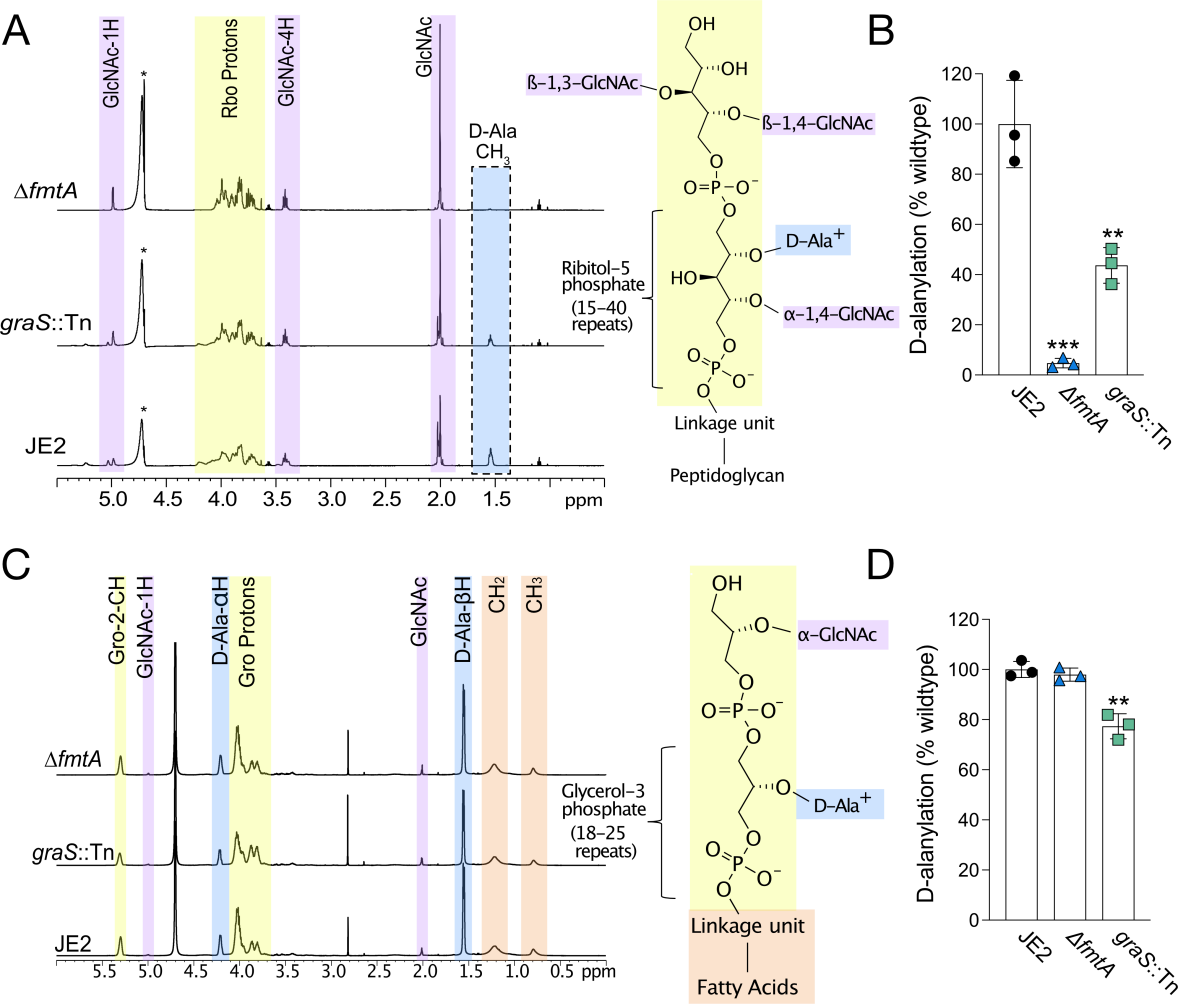
FmtA is essential for wall teichoic acid, but not lipoteichoic acid, D-alanylation. (**A**) ^1^H NMR spectra of WTAs extracted from the MRSA USA300 JE2 (JE2), JE2-*graS*::Tn, and JE2-Δ*fmtA* strains. The artifact at 4.7 ppm is attributed to incomplete water suppression and is marked with an asterisk. (**B**) The ratio of the peak area of the D-Ala methyl group to the peak area of all non-anomeric carbohydrate regions of the NMR spectra. The ratio is represented as a percentage of JE2, where the average ratio from JE2 represents 100%, related to Fig. S4 and Table 1. (**C**) ^1^H NMR spectra of LTA extracted and purified from JE2, JE2-*graS*::Tn, and JE2-Δ*fmtA*. (**D**) The D-Ala to glycerol ratio, represented as a percentage of the wild type, where the average ratio from JE2 represents 100%, related to Fig. S8. Each data point represents a single biological replicate. Error bars represent the standard deviation of the mean. Student’s *t*-test comparing each mutant to the parental strain (JE2) and denoted as **P ≤ 0.01 and ***P ≤ 0.001.

First, the WTAs were extracted from the peptidoglycan sacculus of the different strains using trichloroacetic acid-mediated hydrolysis, as described (14). The purified polymers were characterized using ¹H NMR spectroscopy before (Fig. 2A; Fig. S4) and after (Fig. S5) the addition of NaOH, which induced D-Ala dissociation from the WTA backbone, as previously described (14, 57). The release of D-Ala after the addition of NaOH was confirmed with diffusion-ordered spectroscopy (DOSY) (Fig. S6 and S7). Overall, the ^1^H NMR spectra were consistent with previous studies of *S. aureus* WTA, indicating the samples were homogeneous (14). Previous studies suggest that D-alanylation affects the secondary structure of TAs (30, 38). Notably, the ^1^H NMR peaks for the Rbo-containing non-anomeric protons (4.2–3.1 ppm) were much broader in the parental strain than the ∆*fmtA* mutant (Fig. 2A; Fig. S4), indicating a more dynamic secondary structure. This is consistent with literature demonstrating that D-Ala substitutions form ion pairs with the negatively charged phosphodiester linkages, either upstream or downstream of the D-Ala modification, resulting in a random coiled conformation rather than a rigid extended conformation (30, 38). Following D-Ala hydrolysis (Fig. S5), the peak alignment between the two spectra improved significantly, supporting the notion that D-Ala modifications affect the Rbo-backbone environment and that there are differences between the two strains.

**TABLE 1:** Wall teichoic D-alanylation ratios^*a*^

Strain	Biological replicate	D-Ala:Rbo pH 7.0	D-Ala:Rbo pH > 10.0	D-Ala:GlcNAc pH 7.0	D-Ala:GlcNAc pH > 10.0
JE2	1	0.111	0.108	0.463	0.480
	2	0.099	0.093	0.414	0.423
	3	0.138	0.131	0.572	0.583
JE2-*graS*::Tn	1	0.052	0.053	0.224	0.237
	2	0.042	0.041	0.184	0.182
	3	0.058	0.058	0.253	0.262
JE2-Δ*fmtA*	1	0.0049	0.006	0.022	0.026
	2	0.0036	0.005	0.016	0.021
	3	0.0079	0.011	0.036	0.050

^*a*^ The relative degree of D-alanylation was calculated from the ^1^H NMR spectra by taking the ratio of the integrated area of the D-Ala methyl group to the peak area of the non-anomeric carbohydrate signals, which includes the Rbo backbone. The integral ratio of the D-Ala methyl group to the GlcNAc methyl group was also calculated. These values were collected before (pH 7.0) and after (pH > 10.0) the addition of NaOH. Related to Fig. 2 and Fig. S4.

We determined relative levels of WTA D-alanylation by calculating the ratio of the D-Ala methyl peak area to the GlcNAc methyl peak area (Table 1), using a previously described method (20, 58). To account for potential strain-to-strain differences in GlcNAc modification (15), we also calculated the ratio relative to the Rbo-containing non-anomeric carbohydrate region of the NMR spectra (Table 1; Fig. 2B). This region still incorporates GlcNAc non-anomeric protons and is therefore subject to variations in WTA glycosylation; however, we compared the GlcNAc methyl peak area to the Rbo-containing non-anomeric carbohydrate region and found similar glycosylation levels across the strains: JE2 (0.240 ± 0.002), the *graS*::Tn mutant (0.230 ± 0.002), and the *fmtA* mutant (0.222 ± 0.003). Notably, all of these ratios only offer a comparative assessment of GlcNAc/D-alanylation levels and do not directly quantify the amount of GlcNAc/D-Ala present. Nonetheless, both ratios demonstrate that the *graS*::Tn mutants contain a moderate level of WTA D-alanylation, with a ~56% reduction in the relative degree of D-alanylation compared to the parental strain. This finding is consistent with other studies demonstrating a 46.7% decrease in TA D-Ala content in *graRS* mutants (59). In contrast, the *fmtA* mutant exhibited drastically less WTA D-alanylation (~95% reduction), similar to levels observed in LTA-deficient *S. aureus* (20). These substantial changes in D-Ala content likely contribute to the increased net negative surface charge observed in both mutants. While decreased WTA D-alanylation in the *graS* mutant likely plays a role, it is unlikely to be solely responsible, given the pleiotropic effects of disrupting this TCS and its influence on other key regulators of cell surface charge, such as MprF (49). However, the changes in D-Ala content correlate with the cell surface measurements, with the *fmtA* mutant exhibiting a more negative cell surface and less WTA D-Ala.

Next, we extracted LTA using 1-butanol and further purified it using hydrophobic interaction chromatography (14). We hypothesized that LTA from the ∆*fmtA* mutant would either exhibit increased D-Ala content due to a lack of esterase activity or have equivalent D-alanylation if the parental strain’s LTA is already maximally modified under these growth conditions. The purified polymers were analyzed using ^1^H NMR spectroscopy, demonstrating consistency with previous studies (14) (Fig. 2C; Fig. S8). The ratio of glycerol to D-Ala was calculated as previously described (57) for JE2 (75.6% ± 2.4%), the *fmtA* mutant (74.0% ± 2.0%), and the *graS*::Tn mutant (58.5% ± 3.8%) (Fig. 2D). The measured D-Ala levels in the parental strain corroborate previous reports, indicating that *S. aureus* LTA is approximately 70% D-alanylated (57). As anticipated, the level of D-alanylation was significantly decreased in the *graS* mutant (Fig. 2C; Fig. S8). In contrast, the *fmtA* mutant exhibited comparable levels of LTA D-alanylation to the parental strain, suggesting that LTA D-Ala modifications have reached their threshold in the parental strain at the early exponential stage of the growth cycle (Fig. 2C; Fig. S8). Collectively, these findings suggest that the alterations in cell surface charge observed in *fmtA*-deficient strains are correlated with a significant reduction in WTA D-alanylation.

### Differential spatial localization of surface charge is associated with distinct effects on antimicrobial peptide susceptibility

Given that bacterial surface charge influences susceptibility to cationic antimicrobial peptides (CAMPs), we next examined CAMP susceptibility in the mutants to explore the link between TA D-alanylation and resistance. We began by assessing sensitivity to polymyxin B (PMB), a peptide that typically targets Gram-negative bacteria through electrostatic interactions with the lipid A component of lipopolysaccharide. While PMB efficacy is often limited in Gram-positive bacteria, such as *S. aureus*, increased cell surface negativity can enhance susceptibility (60).

**Fig 3:**
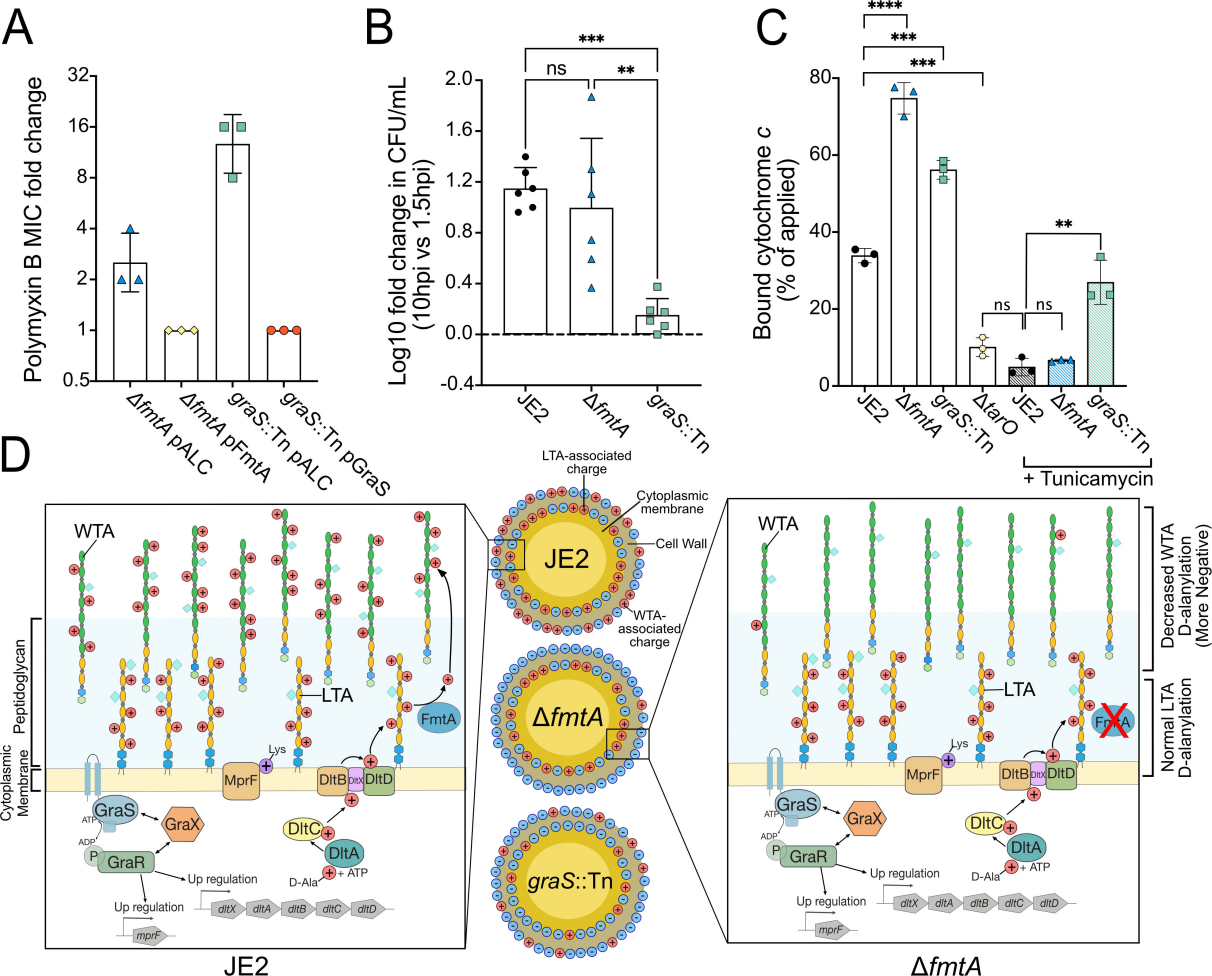
*ftmA* and *graS* differentially impact CAMP susceptibility, macrophage survival, and LTA-associated cell surface charge. (**A**) Polymyxin B minimum inhibitory concentration (MIC) fold change relative to the parental strain (JE2), determined from two technical replicates per biological replicate; complementation with pALC2073 restored susceptibility, while an empty vector served as control (related to Table 2). (**B**) Macrophage (RAW 264.7) survival following gentamicin treatment at 10 and 1.5 h post-infection (hpi), quantified by colony-forming unit (CFU) enumeration. The data represent a single biological replicate from two independent experiments. (**C**) Measuring cytochrome *c* binding to JE2 after tunicamycin treatment; Δ*tarO* served as a WTA-deficient control. Each data point represents a single biological replicate, averaged from three technical replicates. Error bars represent the standard deviation of the mean. Statistical significance was determined by one-way ANOVA with Tukey’s multiple comparisons test and two-tailed unpaired Student’s *t*-test and was denoted as *P ≤ 0.05, **P ≤ 0.01, ***P ≤ 0.001, and ****P ≤ 0.0001. (**D**) Schematic illustrating cell surface charge regulation. The GraXRS upregulates *mprF* and *dltXABCD*, leading to phospholipid lysinylation (purple positive charge) via MprF and D-alanylation of LTA by DltXABCD. Disruption of *graS* reduces membrane and surface charge; FmtA removes D-Ala from LTA for WTA incorporation, increasing negative charge when *fmtA* is absent.

As expected (61), the *graS* mutant—which displays reduced LTA D-Ala content (Fig. 2C) and decreased MprF expression leading to less phospholipid lysinylation (62)—showed increased PMB sensitivity (Fig. 3A). We speculated that the *fmtA* mutant would exhibit a different response since the LTA D-Ala levels remain unchanged (Fig. 2C). Indeed, compared to the parental strain, only the *graS*::Tn mutant exhibited significantly (greater than twofold) increased susceptibility to PMB, and gene complementation restored resistance (Fig. 3A; Table 2).

**TABLE 2:** The impact of teichoic acid D-alanylation on antimicrobial susceptibility^*a*^

Strain	Antibiotic	Antibiotic charge	MIC (μg/mL)	Fold change
JE2 pALC2073	Polymyxin B	+5	128.0	–^*b*^
JE2-∆*fmtA* pALC2073	Polymyxin B	+5	64.0	2
JE2-∆*fmtA* pFmtA	Polymyxin B	+5	128.0	1
JE2-*graS*::Tn pALC2073	Polymyxin B	+5	8.0	16
JE2-*graS*::Tn pGraS	Polymyxin B	+5	128.0	1
JE2 pALC2073	Nisin	+4	512.0	–
JE2-∆*fmtA* pALC2073	Nisin	+4	256.0	2
JE2-∆*fmtA* pFmtA	Nisin	+4	512.0	1
JE2-*graS*::Tn pALC2073	Nisin	+4	256.0	2
JE2-*graS*::Tn pGraS	Nisin	+4	512.0	1

^*a*^ Strains were assessed in biological triplicate against polymyxin B and in technical triplicate against nisin. For each strain, the minimum inhibitory concentration (MIC) was compared to JE2-pALC2073 to determine the fold change. Decreases in resistance of ≥4-fold are bolded. Twofold decreases fall within the acceptable error range and are not considered significant. Antibiotic charge is based on physiological pH.

^*b*^ “–” indicates no value.

We also evaluated susceptibility to the lantibiotic nisin, a positively charged and polycyclic peptide that acts on the cytoplasmic membrane and is dependent on lipid II (63); however, consistent with the literature (61), we observed no change in sensitivity in either strain (Table 2). This finding is likely due to the BraRS (also NsaRS) TCS, which regulates nisin susceptibility by upregulating the BraDE and VraDE ABC transporters (64, 65).

*S. aureus* also encounters CAMPs within macrophages, and GraS is essential for the replication and survival of *S. aureus* in acidified phagolysosomes (Fig. 3B) (66). However, the deletion of *fmtA* did not influence *S. aureus* proliferation within macrophages (Fig. 3B). Together, this suggests that although the relative surface charge of a *fmtA* mutant is more negative (Fig. 1) due to the absence of WTA D-alanylation (Fig. 2), the inner membrane charge remains similar to that of the parental strain, consistent with unchanged levels of LTA D-alanylation.

To investigate further, we used tunicamycin, a WTA synthesis inhibitor (67, 68), to study cell surface charge in a WTA-deficient background. First, we demonstrated that the affinity of cytochrome *c* for a ∆*tarO* mutant lacking WTA was significantly reduced compared to the parental strain (69) (Fig. 3C). This finding suggests that the cell surface of the mutant is more positively charged, likely due to the absence of negatively charged WTA. Similarly, the parental strain treated with subinhibitory tunicamycin concentrations (1 µg/mL) had equivalent cytochrome *c* binding to the ∆*tarO* mutant, indicating that tunicamycin can be used to disrupt WTA, enabling the study of LTAs (Fig. 3C). Like the parental strain, the ∆*fmtA* mutant displayed weak binding to cytochrome *c* following tunicamycin treatment. This finding demonstrates that the cell surface becomes more positively charged when WTA is absent in the ∆*fmtA* mutant, a result consistent with LTAs having comparable D-Ala content to the parental strain (Fig. 3C). In contrast, following tunicamycin treatment, the *graS*::Tn mutant bound more cytochrome *c* than the parental strain and the ∆*fmtA* mutant (Fig. 3C); however, this phenotype may be associated with other factors regulated by the GraRS TCS (Fig. 3D) (49).

In summary, these results indicate that while the *fmtA* mutant exhibits reduced WTA D-alanylation and a decreased net surface charge, LTA D-alanylation remains unaffected. As a result, CAMP susceptibility, macrophage replication, and inner membrane relative surface charge remain unchanged, highlighting the importance of differential spatial localization of surface charge (Fig. 3D) in determining CAMP susceptibility.

### Wall teichoic acid D-alanylation is important for *S. aureus* adhesion

**Fig 4:**
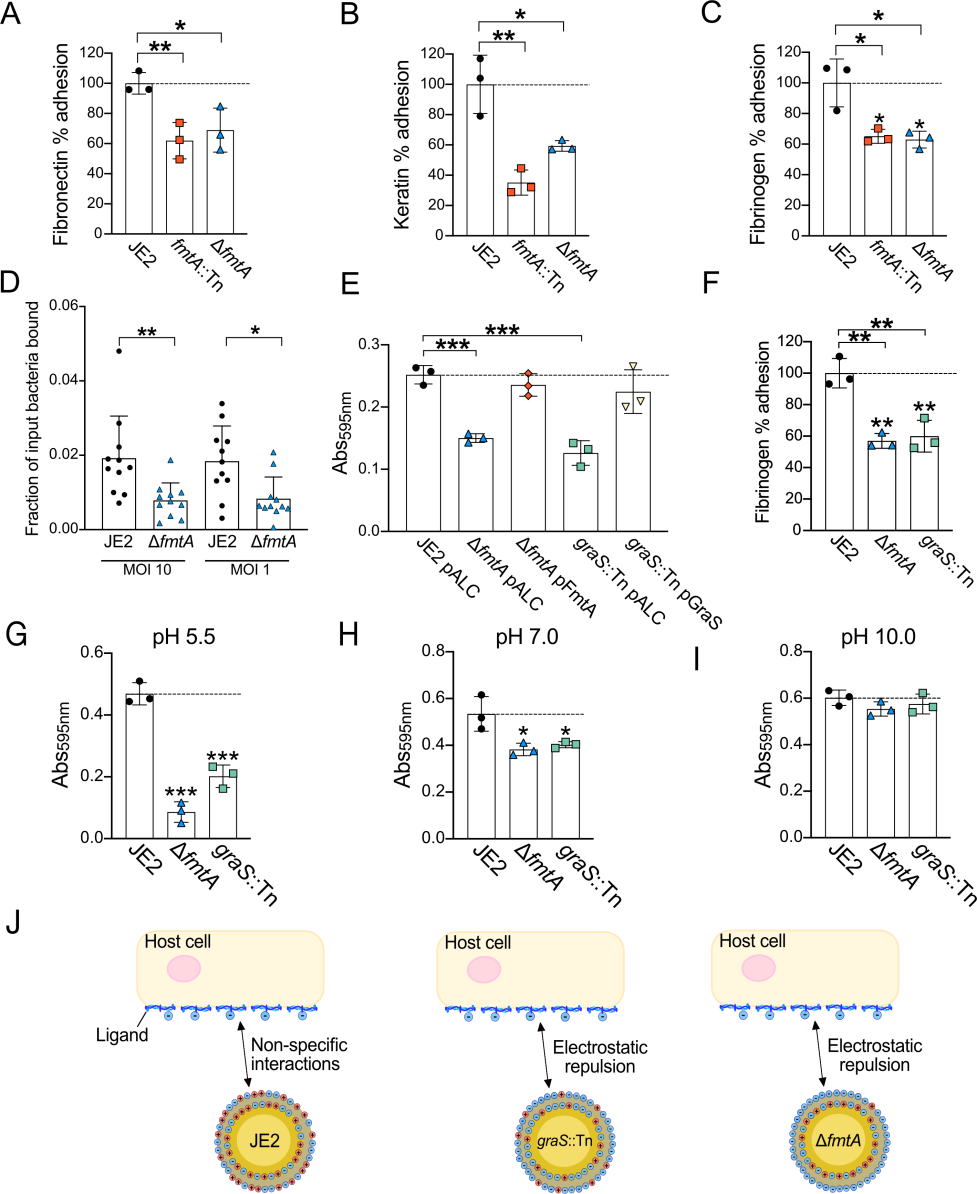
Disruption of teichoic acid D-alanylation impairs *S. aureus* host adhesion. (**A–C**) Adhesion to fibronectin (0.5 µg/well), keratin (0.0625 µg/well), and fibrinogen (0.25 µg/well) was quantified by manual enumeration of JE2 colony-forming units (CFUs). Data are presented as percentages relative to wild-type JE2 (100%). Each data point represents the mean from three technical replicates within a single biological replicate. (**D**) Adhesion to HMEC-1 cells was measured as the fraction of bacteria bound after 10 min at the indicated MOIs. Data are derived from three independent experiments and analyzed by unpaired Student’s *t*-test with Welch’s correction. Disruption of *graS* reduced adhesion to fibrinogen (crystal violet staining [**E**] and CFU enumeration [**F**]), a phenotype restored by complementation using the pALC vector (**E**). Adhesion was also assessed following incubation in tryptic soy broth at pH 5.5 (**G**), 7.0 (**H**), and 10.0 (**I**). (**J**) Schematic illustrating how increased cell surface negative charge, resulting from reduced D-alanylation, can decrease adhesion through electrostatic repulsion. Unless otherwise stated, error bars represent the standard deviation of the mean; *P*-values were calculated using a two-tailed unpaired Student’s *t*-test comparing each mutant to JE2 (*P ≤ 0.05, **P ≤ 0.01, and ***P ≤ 0.001).

Building upon our previous observations that disrupting *fmtA* reduces *S. aureus* adhesion to host ligands (42), we sought to confirm these findings using two distinct adhesion detection methods. We observed significantly reduced adhesion of the ∆*fmtA* mutant to fibronectin, keratin, and fibrinogen (Fig. 4A through C; Fig. S9). Additionally, *S. aureus* lacking *fmtA* exhibited decreased binding to human endothelial cells (HMEC-1), highlighting the physiological significance of this phenotype (Fig. 4D). Consistent with the ∆*fmtA* mutant, disruption of *graS* also reduced *S. aureus* affinity for fibrinogen (Fig. 4E and F). Restoring the *graS* gene through complementation reversed these phenotypes (Fig. 4E). These adhesion defects persisted under acidic and neutral pH conditions (Fig. 4G and H) but were absent at alkaline pH (Fig. 4I), suggesting a direct link between the phenotype and D-Ala content within WTAs.

Finally, since high-affinity interactions between *S. aureus* and host ligands require the surface display of cell wall-anchored (CWA) proteins such as the fibronectin-binding proteins A and B (FnbpA/B) (2, 42, 70), we also measured the abundance of the FnbpA/B on the surface of whole cells using an enzyme-linked immunosorbent assay (ELISA)-based approach (70). FnbpA/B surface abundance was unchanged in ∆*fmtA* and *graS*::Tn (Fig. S10). These data suggest that the adhesion defects are likely due to changes in cell surface charge rather than the display of CWA proteins (Fig. 4J).

**Fig 5:**
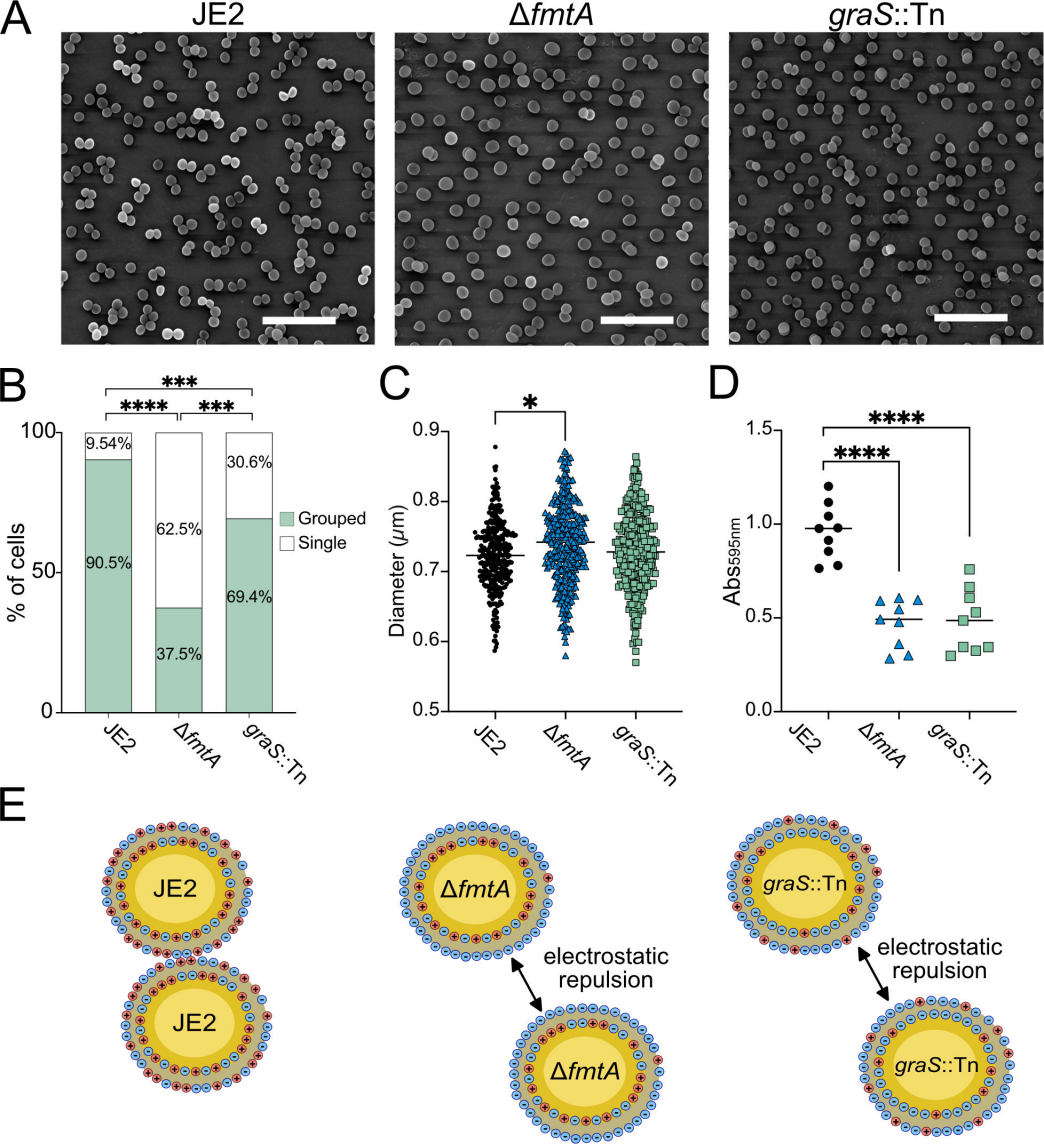
Disruption of teichoic acid D-alanylation impairs cell aggregation and biofilm formation. (**A**) Scanning electron microscopy analysis of MRSA USA300 JE2 (JE2), JE2-∆*fmtA,* and JE2-*graS*::Tn. Scale bars represent 5 µm. (**B**) Cell aggregation (**A**) was quantified by counting total cells in a consistent dimension (~200 cells) for three biological replicates per strain. Single cells versus grouped (≥2) cells were counted to determine the percentage of cell aggregation. (**C**) Cell diameter (**A**) (*n* = 100 cells) was measured using ImageJ for three biological replicates. Each data point represents a single cell measurement. Lines represent the average cell length for each strain. (**D**) Biofilm formation (Abs_595nm_) in the presence of citrate. Each data point represents a biological replicate assessed using 18 technical replicates. Lines represent the average Abs_595nm_ for each strain. *P*-values were calculated using the two-tailed unpaired Student’s *t*-test comparing strains and were denoted as *P ≤ 0.05, **P ≤ 0.01, ***P ≤ 0.001, and ****P ≤ 0.0001. (**E**) Schematic depicting cell-cell interactions. When the cell surface is more negatively charged, electrostatic repulsion impairs cell clumping.

We then investigated cell aggregation (71) and biofilm formation since TA D-Ala content influences these processes in Gram-positive organisms (72–74). Scanning electron microscopy (SEM) revealed that *fmtA* and *graS* are required for bacterial cell-cell adhesion and aggregation (Fig. 5A and B). We also noted the Δ*fmtA* mutant exhibited a slightly increased cell diameter (Fig. 5C), likely due to decreased cell wall density following the loss of WTA D-Ala esters (38, 75). Consistent with previous reports (27, 72, 73), both mutants formed significantly less citrate-induced biofilm compared to the parental strain (Fig. 5D). In conclusion, these results demonstrate that WTA D-alanylation is important for host adhesion, cell aggregation, and biofilm formation. Based on the findings of this study, we propose that impaired adhesion is due to an altered surface charge, which impacts electrostatic interactions crucial during the early stages of adhesion (Fig. 5E) (43, 44).

## DISCUSSION

*S. aureus* utilizes the TA D-alanylation pathway to regulate cell surface charge, influencing processes crucial for survival in the host, including biofilm formation, surface attachment, antimicrobial susceptibility, and macrophage survival (66, 73). While the Dlt pathway plays a well-established role in LTA D-alanylation (18), the mechanisms governing WTA D-alanylation remain unclear. Early pulse-chase experiments with [C^14^]-alanine showed that D-Ala is initially incorporated into LTA, and subsequent decreases in LTA radioactivity correlated with increases in WTA radioactivity—the first evidence suggesting that LTA serves as a source for WTA D-Ala (24). This model was further supported by the drastic reduction of WTA D-alanylation in an LTA-deficient mutant, suggesting that transiently membrane-bound WTAs are inefficient D-Ala acceptors, highlighting LTA’s critical role (20).

Here, we provide further evidence supporting the notion that LTA D-Ala serves as a source for WTA D-alanylation. We initially investigated this phenomenon because *fmtA*-deficient *S. aureus* exhibited reduced adhesion to host ligands (42), which we hypothesized was due to alterations in cell surface charge impacting electrostatic interactions between the bacterium and host ligand (43, 44). Exploring this phenotype uncovered a biologically important finding: FmtA is essential for WTA D-alanylation (Fig. 2).

While previous work has proposed an alternative model suggesting increased WTA D-alanylation in an *fmtA* mutant (29), we present comprehensive NMR spectra from three independent replicates of WTA and LTA extracted from MRSA USA300 JE2 clone LAC grown to the early exponential phase (Fig. 2; Fig. S4 and S8), allowing for a detailed analysis of these factors. Our findings are further supported by charge-based experiments (Fig. 1) and phenotypic assays that align with the widely accepted model that LTA serves as the primary donor for WTA D-alanylation. Ultimately, our data suggest that FmtA removes D-Ala from LTAs, facilitating transfer to WTA, as depicted in Fig. 3D. While previous studies have ruled out direct transesterase activity for FmtA (29), we propose that it likely functions in concert with other enzymes within the cell envelope due to energetic constraints, preventing D-Ala release into the environment. Further research is needed to elucidate the precise mechanisms governing this transfer comprehensively and reaffirm FmtA’s role in D-Ala removal from LTA.

As expected, variations in TA D-alanylation significantly impacted several phenotypes associated with host colonization and infection, including host adhesion (Fig. 3 to 5). In particular, host adhesion, biofilm formation, and cell-cell interactions were significantly impacted, which we attribute to increased electrostatic repulsion between negatively charged surfaces (Fig. 4J) (43). This is consistent with the fact that all of the host ligands evaluated are negatively charged at physiological pH (76–79).

Notably, differential spatial localization of surface charge seems to confer distinct effects on antimicrobial peptide susceptibility (Fig. 3). This finding highlights that variations in surface charge distribution, rather than overall charge alone, play a crucial role in determining resistance to antimicrobial peptides. Although D-alanylation of LTAs influences surface charge near the cell membrane, LTAs and WTAs occupy distinct spatial locations within the cell envelope; WTAs are attached to the peptidoglycan cell wall, protruding from the cell surface, while LTAs are membrane-bound and buried below the cell wall (7–9, 80). This separation likely explains how localized charge differences can differentially impact antimicrobial peptide interactions with *S. aureus*. Beyond charge-based interactions influencing CAMP susceptibility, previous studies have also shown that D-alanylation impacts LTA secondary structure. This, in turn, affects peptidoglycan density and the accessibility of the membrane (30, 38, 75, 81). Since the D-alanylation levels of LTAs extracted from the *fmtA*-deficient mutant remain unchanged, CAMP susceptibility was also unaffected; however, we acknowledge that other cellular factors could be contributing to this phenomenon, which should be the focus of future studies.

In conclusion, our findings demonstrate that FmtA plays a critical role in WTA D-alanylation, strongly suggesting its involvement in facilitating the transfer of D-Ala from LTA to WTA. While the precise mechanisms governing this transfer remain to be elucidated, unraveling them may lead to the identification of novel antivirulence targets and the development of innovative strategies to combat the escalating threat of MRSA.

## MATERIALS AND METHODS

### Bacterial strains, plasmids, and growth conditions

Unless otherwise stated, CA-MRSA USA300 strain JE2 (82) was used as the parental strain in this study. Transposon mutants of interest were obtained from the Nebraska transposon mutant library (NTML) (46), and the presence of the transposon within the gene of interest was confirmed via PCR using gene- and transposon-specific primers. The NTML (46) and the NTML genetic toolbox (82) were obtained through BEI Resources (www.beiresources.org) from the Network on Antimicrobial Resistance in *S. aureus* Repository. We generated *fmtA*:Tn mutants in the MRSA USA300 strain AH1263 and the laboratory *S. aureus* strain RN4220 by transducing the transposon from the USA300 JE2-Δ*fmtA*:Tn mutant (NTML) using phage 80α (42). Successful insertion of the transposon was confirmed via PCR using gene-specific primers. All *S. aureus* strains were routinely propagated in tryptic soy broth (TSB; Wisent Inc.) at 37°C with aeration at 220 rpm unless otherwise stated. The pALC2073:*fmtA* plasmid was generated by amplifying the *fmtA* gene from JE2 genomic DNA using the FmtA_Fwd_Comp and FmtA_Rvs_Comp primers, followed by ligation into pALC2073 using the *Kpn*I and *Eco*RI restriction sites. Successful ligation was confirmed using whole plasmid sequencing (Plasmidsaurus).

We routinely employed *Escherichia coli* IM08B (BEI Resources) as a cloning host, and *S. aureus* RN4220 (83, 84) as an intermediate host before electroporation into JE2. All *E. coli* strains were cultured in Lysogeny broth (BioShop Canada) at 37°C with aeration at 220 rpm unless otherwise specified. Antibiotics were used for selection at the following concentrations: ampicillin (100 µg/mL), chloramphenicol (10 µg/mL), and erythromycin (5 and 10 µg/mL). A complete list of strains, vectors, and primers is provided in Tables S1 and S2, respectively.

### Allelic exchange

Allelic exchange was performed as previously described (45, 70). Briefly, the upstream and downstream flanking regions of the *fmtA*, *spa*, and *sbi* genes were amplified using the following primers: PrimerA_FmtA, PrimerB_FmtA, PrimerC_FmtA, PrimerD_FmtA, and PrimerA_Spa, PrimerB_Spa, PrimerC_Spa, PrimerD_Spa, and PrimerA_Sbi, PrimerB_Sbi, PrimerC_Sbi, PrimerD_Sbi (Table S2). Amplicons were recombined into pJB38 (82), and the plasmids were generated in *E. coli* DH5α at 30°C, followed by passage through *E. coli* IM08B or *S. aureus* RN4220 at 30°C. The plasmids were then transformed into MRSA USA300 JE2 strains of interest using electroporation, followed by incubation at 30°C. For recombination, the strains harboring the plasmid of interest were streaked from their respective glycerol stocks onto TSA with chloramphenicol (10 µg/mL) and were incubated at 43°C overnight to initiate recombination. Large colonies, indicative of a single recombination event, were propagated again overnight at 43°C. An isolated large colony was then inoculated into 3 mL of TSB and incubated overnight at 30°C. The bacterial cultures were then diluted 1:500 into TSB for four consecutive days to mediate plasmid excision and further recombination. To select for curing of the plasmid, the cells were plated onto TSA with 2, 1.5, 1, and 0 µg/mL anhydrotetracycline (Thermo Scientific). Colonies were patched onto TSA and TSA with chloramphenicol (10 µg/mL). Colonies that only grew in the absence of the antibiotic indicated the successful removal of the plasmid. Gene deletions were confirmed using Sanger sequencing (Advanced Analysis Centre, University of Guelph).

### Reverse transcription-quantitative PCR to measure *dlt* expression

A single colony was inoculated into 3 mL of TSB for each strain in biological triplicate, followed by propagation at 37°C with aeration at 220 rpm for 16 h. The following day, total RNA was extracted from the overnight cultures using an RNeasy Mini Kit (Qiagen) according to the manufacturer’s guidelines. Briefly, the cells were harvested by centrifugation and resuspended in 100 µL of solution A (TE buffer and 50 mg/mL lysozyme) and 100 µg/mL lysostaphin. The cell cultures were then incubated at 37°C for 30 min to lyse the bacterial cells. Following incubation, 350 µL of RLT buffer (Qiagen) and 3.5 µL of β-mercaptoethanol were applied and gently mixed. A volume of 250 µL of 100% ethanol was then added, and the samples were transferred to RNeasy columns (Qiagen). The quality and quantity of RNA were determined using a Nanodrop 2000 Spectrometer (Thermo Fisher). cDNA was synthesized from 750 ng of total RNA (eluted in DEPC-treated RNase- and Nuclease-free water [Invitrogen by Thermo Fisher Scientific]). A Turbo DNA-free Kit (Invitrogen by Thermo Fisher Scientific) was used according to the manufacturer’s guidelines to remove contaminating genomic DNA from the RNA samples. cDNA was synthesized using the Superscript IV First-Strand Synthesis System Kit (Invitrogen) primed with random hexamers according to the manufacturer’s guidelines. A negative reverse transcriptase (−RT) control was prepared to ensure that there was no significant genomic DNA contamination. All samples were stored at −20°C.

Primers (Table S2) were designed to amplify ~100 bp products for the target gene (*dltA*) and the reference gene (*rpoD*) using the PrimerQuest Tool (IDT). *rpoD* was selected since it is expressed at a similar level over various experimental conditions and is proposed to be a good reference for quantifying relative gene expression in *S. aureus* (85). Initial experiments were performed to optimize cDNA concentrations for qPCR using the Powertrack SYBR Green Master Mix Kit (Thermo Fisher Scientific) according to the manufacturer’s guidelines. A 5-point, 10-fold, serial dilution of cDNA with a concentration range of 9.3750 –0.0009 ng/µL was tested using an optimized primer concentration of 0.5 µM, and a Powertrack SYBR Green Master Mix according to the manufacturer’s guidelines. Optimization was based on amplification. A single amplicon was indicated by a melt curve with a single peak, and amplification of the same cDNA fragment was indicated by a mean ± 0.2°C difference in the melt temperature of samples with the same target. The absence of amplification in the no-template control indicated that there was no contamination. The following quality control parameters based on Thermo Fisher guidelines were used to ensure the standard curve was acceptable: efficiency of 90%–100%, slope between −3.58 and −3.10, and coefficient of correlation (*R*^2^) > 0.99. Briefly, 5 µL of cDNA (diluted 1:2,000 in DEPC-treated RNase- and nuclease-free water) was applied to optical 96-well plates (Thermo Fisher Scientific). A volume of 15 µL of master mix with primers (0.5 µM final concentration) was applied to each well. Real-time qPCR reactions were carried out in a final volume of 20 µL. Three technical replicate reactions were prepared for each biological replicate. A control lacking a template was included for each primer set, and the −RT control to ensure that no contaminating genomic DNA was present in the original RNA samples. PCR conditions for qPCR were as follows: an initial hold at 50°C for 2 min, denaturation at 95°C for 10 min, followed by 40 consecutive cycles of 15 s at 95°C and 1 min at 60°C. QuantStudio3 (Applied Biosystems by Thermo Fisher Scientific) was used for real-time qRT-PCR, and Thermo Fisher QuantiGene Plex Data Analysis (version 2.6.2) was used for plate setup and analysis. Relative quantification was carried out, and relative fold gene expression was calculated using the ∆∆Ct Method (Livak Method) (85).

### *S. aureus* surface charge measurement using cytochrome *c*

Relative bacterial cell surface charge was determined by quantifying binding of *S. aureus* to cationic cytochrome *c*, as previously described with minor modifications (50). Briefly, saturated overnight cultures in TSB were diluted 1:100 in TSB and incubated at 37°C with aeration (220 rpm) until an OD_600nm_ of 1.0 was achieved, unless otherwise stated. When necessary, the TSB was supplemented with 1 µg/mL of tunicamycin, 100 mM MES (pH 5.5), 5 mM HEPES (pH 7.0), or 50 mM bis-tris propane (BTP) (pH 10.0). The cells were harvested by centrifugation at 4,000 × *g* for 10 min at 4°C, washed with phosphate-buffered saline (PBS) (pH 7.4), and resuspended in PBS (pH 7.4) to an OD_600nm_ of 3.0. When necessary, the PBS was supplemented with 100 mM MES (pH 5.5), 5 mM HEPES (pH 7.0), or 50 mM BTP (pH 10.0). Following standardization, 1 mL aliquots were centrifuged (6,000 × *g* for 2 min) and resuspended in 500 µL of 0.5 mg/mL cationic cytochrome *c* protein from equine heart (Sigma) solubilized in sterile water or, when necessary, 100 mM MES (pH 5.5), 5 mM HEPES (pH 7.0), or 50 mM BTP (pH 10.0). The cells were then incubated with shaking (220 rpm) for 15 min at 37°C. For assessing LTA-related surface charge, the cytochrome *c* incubation was extended from 15 min to 1 h. The cells were then harvested by centrifugation (6,000 × *g* for 2 min), and 100 µL of the supernatant was applied to a 96-well round-bottom tissue culture microtiter plate (VWR 10861-564; Avantor) in technical triplicate. The Abs_410nm_, representing the amount of unbound cytochrome *c,* was measured using a BioTek Synergy H1 microplate reader. To calculate the percentage of bound cytochrome *c*, the Abs_410nm_ of the sample was subtracted from the Abs_410nm_ of the original 0.5 mg/mL cytochrome *c* solution.

### Chemical force microscopy

The bacterial strains were cultured on TSA plates overnight at 37°C. A single colony was incubated in 10 mL TSB overnight at 180 rpm at 37°C. The cells were harvested by centrifugation (2,000 × *g* for 5 min), washed once with TSB, diluted in the same medium to an optical density at 600 nm of 0.05, and propagated to the exponential phase of growth (OD_600nm_ = 0.3). JE2-*graS*::Tn cells were cultured in TSB supplemented with erythromycin (5 µg/mL). Afterward, the cells were harvested by centrifugation and washed twice with 20 mM HEPES (pH 7.0). The cell suspension was diluted 1:100 and placed on TPP™ polystyrene Petri dishes for 15 min, then gently rinsed with 20 mM HEPES before AFM measurements. AFM cantilevers (PNP-TR-Au, Nanoworld) were functionalized with alkanethiols terminated with carboxyl groups, as previously described (54). The cantilevers were first cleaned for 15 min by ultraviolet (UV)/ozone treatment (Jelight Co., Irvine, CA, USA) and then immersed overnight in 1 mM 16-mercaptohexadecanoic acid (Sigma) in ethanol. The cantilevers were then rinsed and stored in 20 mM HEPES solution (pH 7.0) before experiments. Chemical force microscopy experiments were performed in 20 mM HEPES (pH 7.0) at room temperature using a JPK NanoWizard 3 NanoScience AFM instrument. Cantilevers were calibrated using the thermal noise method, yielding spring constants in the 0.043–0.054 N/m range. Adhesion between carboxylated cantilevers and single bacterial cells was spatially mapped by recording force-distance curves in force-volume mode (32 × 32 pixels) in 500 × 500 nm^2^ regions on top of the cells, applying a force setpoint of 0.25 nN and a constant approach/retract speed of 1 µm/s. Force data were analyzed with JPK Data Processing software (version 8.0.144). For each cell probed, adhesion forces were plotted as histograms, and mean force values were extracted by fitting the data with a Gaussian function. Statistical analyses were performed with Origin software (OriginPro 2021).

### Extraction and purification of TAs

LTA and WTA were extracted and purified as previously described (14), with minor modifications. Saturated overnight cultures were diluted (1:100) in 10 L of TSB and incubated at 37°C with aeration (220 rpm) until an OD_600nm_ of 1.0 was achieved. The cells were harvested by centrifugation (4,000 *× g* for 15 min at 4°C), resuspended in 100 mL of 2 M NaCl, and lysed with 0.1 µM ceramic beads (OMNI International) using a FastPrep Homogenizer (MP Biomedicals) (three cycles consisting of six rounds of 4 m/s for 40 s) or a Bead Ruptor Elite (OMNI) (four cycles consisting of 10 rounds of 3.1 m/s for 40 s), with incubation on ice in between the 40 s cycles. After each cycle, the lysate was clarified by centrifugation at 5,500 *× g* for 10 min at 4°C. The cell wall was isolated by centrifugation (14,000 × *g* for 15 min at 4°C) and then washed once in PBS before overnight storage at −20°C. The membrane containing lysate was transferred into clean tubes, and the membrane fraction was collected by centrifugation (30,000 × *g* for 1 h at 4°C) before storage at −80°C.

To extract WTA, the cell walls were washed three times in 0.5% sodium dodecyl sulfate, three times in Milli-Q H_2_O, and then resuspended in 30 mL of Milli-Q H_2_O, followed by incubation at 60°C with shaking (220 rpm) for 30 min. The cell wall was then isolated by centrifugation (14,000 × *g* for 15 min at 4°C), washed in Milli-Q H_2_O, and resuspended in 0.2 mg/mL trypsin (Sigma) in 15 mM Tris-HCl (pH 7.0) at 37°C with shaking (220 rpm) for 18 h.

After trypsin digestion, the cell wall was isolated by centrifugation and washed successively with 15 mM Tris-HCl (pH 7.0), 15 mM Tris-HCl (pH 7.0) with 1 M NaCl, 15 mM Tris-HCl (pH 7.0), and then three times with Milli-Q H_2_O. The pellet was then resuspended in 10 mL of 10% trichloroacetic acid (TCA) (wt/vol) and incubated at 4°C for 18 h with constant gentle agitation using a nutating shaker. After TCA-mediated hydrolysis, the peptidoglycan was removed by centrifugation at 5,500 *× g* for 45 min at 4°C, and the supernatant was combined with 1 mL 3 M NaOAc (pH 5.1) and 30 mL of 95% ice-cold ethanol, followed by incubation overnight at −20°C to precipitate the WTAs. The WTAs were collected by centrifugation at 16,000 *× g* for 15 min and were then resuspended in 2 mL of 95% ethanol (Commercial alcohol). The WTAs were then washed five times in 500 µL of 95% ethanol. The ethanol was removed by sublimation under reduced pressure before NMR analysis.

To extract LTA, membrane pellets were washed once in 30 mL resuspension buffer (50 mM sodium citrate, pH 4.7). The membrane was then resuspended in 70 mL of resuspension buffer and 50 mL of 1-butanol. Samples were incubated at 37°C for 45 min with shaking (220 rpm) and subsequently underwent centrifugation (30,000 × *g* for 1 h at 4°C) to pellet insoluble debris and separate the organic and aqueous phases. The aqueous phase containing LTA was collected and underwent two 6 h rounds of dialysis (Spectrum Laboratories; 3500 MWCO) against the resuspension buffer to remove residual butanol before lyophilization, yielding crude LTA. The crude LTA was fractionated using an octyl-Sepharose hydrophobic interaction column (Cytiva HiPrep Octyl FF 16/10). A linear gradient of isopropanol (15%–65% isopropanol [vol/vol] and 50 mM sodium citrate, pH 4.7) was used to elute LTA. Western immunoblotting using a gram-positive bacteria LTA monoclonal primary antibody (Thermo Fisher Scientific) (1:50 in 5% [wt*/*vol] skim milk in TBS + 0.1% [vol/vol] Tween-20] and a rabbit anti-mouse IgG (H + L) HRP-conjugated secondary antibody (Invitrogen) (1:3,000 in 5% [wt/vol] skim milk in TBS + 0.1% [vol/vol] Tween-20) was conducted to identify LTA-containing fractions. LTA-containing fractions were pooled and subjected to extensive dialysis against Milli-Q H_2_O using a Float-A-Lyzer G2 dialysis device with a 0.5–1.0 MWCO (Spectrum Laboratories) before lyophilization.

### ^1^H NMR analysis of TA D-Ala

^1^H NMR experiments were performed at the University of Guelph NMR Centre using a 600 MHz Bruker AVANCE III spectrometer equipped with a 5 mm “TCI” cryoprobe. The WTA samples were dissolved in 60 µL of 100 mM sodium phosphate buffer (pH 7.1) and 540 µL deuterium oxide (Cambridge Isotope Laboratories) and transferred to a 5 mm NMR tube (Bruker) immediately before collecting each spectrum. Subsequently, 30 µL of 1 M NaOH was then added to liberate D-Ala from the WTA backbone. Spectra were collected using the pulse program *zgpr*, with 16 scans per experiment, a relaxation delay of 5.00 s, and an acquisition period of 5.45 s. The optimal pulse width was calibrated before each experiment, and the sample temperature was maintained at 298 ± 1 K. Chemical shift referencing was performed automatically via the spectrometer lock. The LTA samples were prepared in the same manner, except that the sodium phosphate buffer was prepared in pure deuterium oxide. Spectra collected before the addition of NaOH were obtained using the pulse program *zg30*, with the other parameters specified above.

The degree of D-alanylation of LTA samples was determined from the ^1^H NMR spectra acquired at pH 7.0, as previously described (57)*.* Specifically, the ratio of the integrated area of the D-Ala methyl signal (1.60–1.42 ppm) to the total glycerol signal (5.3 and 4.10–3.75 ppm) was calculated. The glycerol integral was corrected by subtracting the contribution of five protons from overlapping GlcNAc resonances in the 4.10–3.75 ppm region. This corrected ratio was multiplied by 5/3 to account for the stoichiometric ratio of the D-Ala methyl resonance (3H) relative to five glycerol protons per repeating unit of the backbone.

It was not possible to adopt this approach to the WTA samples due to the nature of their backbone. Instead, an NMR integration ratio was calculated from the ^1^H NMR spectra acquired at pH 7.0 by taking the ratio of the integrated area of the D-Ala methyl signal (1.60–1.42 ppm) to the integrated area of the GlcNAc methyl signal (2.15–1.85 ppm) (20). Since GlcNAc can vary across strains, the ratio of the integrated area of the D-Ala methyl signal (1.60–1.42 ppm) to the integrated area of the non-anomeric carbohydrate signals (4.2–3.1 ppm) was also determined. The non-anomeric carbohydrate area was corrected by subtracting the contribution of the D-Ala CH proton at 3.6 ppm. The NMR integration ratios across all strains and replicates were then normalized by dividing the average NMR integration ratio for all JE2 replicates.

DOSY spectra were recorded using the *ledbpgppr2s* and *ledbpgp2s* pulse sequences (86). The gradient pulse length was 1.9 ms for WTA and 2.5 ms for LTA, the delay between the “encoding” and “decoding” gradient pulse pairs was 100 ms for WTA and 250 ms for LTA, the gradient stabilization delay was 0.2 ms, and the “LED” period was 5 ms. The gradient strength was increased quadratically in 10–32 steps from 5% to 95% of maximum gradient output, corresponding to 2.41 to 45.7 G/cm (accounting for the smoothed sine-bell amplitude profile of the gradient pulses). Eight transients were collected at each gradient strength, with a 5 s relaxation delay between scans, for a total experiment time of roughly 9–27 min (depending on the number of gradient steps). Spectra were processed in Bruker TopSpin 4.5.0, optimizing a single diffusion component at each chemical shift value.

### Antimicrobial susceptibility testing

Minimum inhibitory concentrations were determined using the CLSI broth microdilution method (87–89). Briefly, glycerol stock strains were applied to TSA and incubated overnight at 37°C. Single colonies were suspended in sterile 0.85% (wt/vol) NaCl to an OD_600nm_ of 0.1 and then diluted 1:100 in cation-adjusted Mueller Hinton II Broth (MHB) (BD Difco). In a round-bottom, untreated 96-well microtiter plate (VWR), the compounds were serially diluted twofold in 50 µL of MHB, followed by the addition of 50 µL of the diluted cell suspension. The plates were incubated at 37°C with aeration at 900 rpm for 18 h (Multitron Shaker, Infors HT). Following incubation, the plates were equilibrated to room temperature (22°C), and the OD_600nm_ was measured using a BioTek Synergy H1 microplate reader.

### Macrophage infection assays

RAW 264.7 macrophages were maintained, passaged, and infected, as previously described (66). In brief, to infect macrophages with *S. aureus*, 1 day before infection, RAW 264.7 cells were scraped in RPMI+ with 5% (vol/vol) FBS and seeded into individual wells of tissue culture-treated 12-well plates to yield a density of ~6.0 × 10^5^ to 8.0 × 10^5^ macrophages per well the following day. At this time, individual colonies of *S. aureus* were inoculated into 5 mL of TSB and cultured overnight at 37°C with aeration at 225 RPM. On the day of infection, the bacteria were subcultured at a dilution of 1:100 in 2 mL of sterile TSB buffered with 5 mM HEPES at pH 7.0.

After 2 h of growth, the bacteria were washed once with sterile saline and diluted into serum-free RPMI to yield an infection at an MOI of 10. The bacteria were applied to individual wells containing RAW macrophages, and phagocytosis was synchronized by centrifugation at 277 *× g* for 2 min. The tissue culture plate containing RAW macrophages and bacteria was then incubated for 30 min at 37°C in a 5% CO_2_ humidified tissue culture incubator. After 30 min, the medium was aspirated and replaced with fresh serum-free RPMI containing gentamicin at 125 µg/mL. After 1 h of treatment with gentamicin, the infected macrophages were washed twice with 1 mL of sterile PBS, and for each replicate, one well was lysed in 0.5 mL sterile Triton X-100 for the manual enumeration of bacteria at 1.5 h post-infection. To the remaining wells, 1 mL of RPMI with 5% (vol/vol) FBS without antibiotics was applied, and the plate was incubated at 37°C in the same tissue culture incubator. Macrophage lysates were drop-plated onto TSA and incubated at 37°C. At 10 h post-infection, the tissue culture medium was aspirated, and extracellular bacteria were harvested by centrifugation. At the same time, wells containing adhered macrophages were lysed as described above, and the lysate was used to resuspend any bacterial pellets after centrifugation to obtain the total bacterial count for each well. Again, these lysates were drop-plated onto TSA and incubated at 37°C. The fold change was calculated for each biological replicate by dividing the bacterial colony-forming units at 10 h post-infection by the bacterial counts obtained at the 1.5 h post-infection time point, followed by calculation of the Log10. This fold change represents the ability of the macrophages to restrict *S. aureus* growth, where Log10 equal to 0 represents no change in CFU (i.e., no growth).

### Crystal violet adhesion assays

The staining of adhered bacterial cells using crystal violet was carried out as previously described (42). Briefly, Nunc MaxiSorp microtiter plates (Thermo Scientific) were coated with 100 µL of host ligands (fibrinogen, fibronectin, or keratin) and incubated at 4°C for 18 h. Fibrinogen (Millipore Sigma) was reconstituted in phosphate-buffered saline with 1.7 mM CaCl_2_ (pH 7.4). Fibronectin (Millipore Sigma) was diluted in PBS lacking CaCl_2_ (pH 7.4).

Keratin (Millipore Sigma) was diluted in a carbonate buffer (15 mM Na_2_HCO_3_ and 35 mM NaHCO_3_, pH 9.6). The following day, unbound host ligands were removed using an aspirator and washed once with 300 µL of PBS (with or without 1.7 mM CaCl_2_) using a BioTek 405 microplate washer. The plates were blocked with 300 µL of 2% bovine serum albumin (BSA) (HyClone) solubilized in PBS (with or without 1.7 mM CaCl_2_) for 1 h at room temperature. Between each of the following steps, the plates were washed three times with 300 µL of PBS (with or without 1.7 mM CaCl_2_). Overnight cultures were prepared in TSB for each strain in biological triplicate, as described above. The following day, saturated overnight cultures were diluted 1:100 in TSB and propagated at 37°C with shaking (220 rpm). For pH-dependent adhesion experiments, propagation occurred in TSB supplemented with 100 mM MES buffer (pH 5.5), 5 mM HEPES (pH 7.0), or 50 mM bis-tris propane (pH 10.0). Bacterial cultures were propagated to a previously optimized OD_600nm_ value based on the expression of the major adhesin responsible for adhesion to the host ligand of interest (0.3 for fibronectin, 0.6 for keratin, and 1.0 for fibrinogen) (42). A volume of 100 µL of each culture was applied to the host ligand-coated microtiter plate wells. The plates were incubated for 5 min with shaking (900 rpm) and then for 1 h with gentle agitation (100 rpm). The plate lids were removed for the final 20 min of incubation, and cultures were irradiated under ultraviolet light. After incubation and washing the plates as described, 100 µL of 0.5% crystal violet (wt*/*vol, in 20% methanol [vol/vol]) was applied to each well and incubated for 4 min at room temperature. The plates were then washed three times with 400 µL of PBS with or without Ca^2+^. The crystal violet was solubilized in 7% acetic acid, and the Abs595_nm_ was measured using a BioTek Synergy H1 microplate reader.

### Colony-forming unit adhesion assays

The manual enumeration of CFUs was performed as previously described (70).

NuncMaxiSorp microtiter plates (Thermo Scientific) were coated with either 0.25 µg/well fibrinogen (Millipore Sigma), 0.5 µg/well fibronectin (Millipore Sigma), or 0.0625 µg/well keratin (Millipore Sigma), incubated overnight, washed, and blocked as described above.

Overnight cultures were prepared in biological triplicate for each strain and diluted 1:100 in TSB, followed by propagation at 37°C with shaking (220 rpm). Once the desired OD_600nm_ was achieved, 60 µL of bacterial culture and 60 µL of PBS (pH 7.4) were applied to the host ligand-coated microtiter plates. For each biological replicate, three technical replicates were performed. To enumerate the CFUs applied, 20 µL of culture was immediately removed and diluted 1:10 in sterile 0.85% (wt/vol) NaCl for manual enumeration of CFUs. The remaining 100 µL of bacterial cells in the microtiter plate wells was incubated with the host ligands for 5 min with vigorous agitation (900 rpm), followed by 1 h with gentle shaking (100 rpm) at room temperature. The plates were then washed as previously described. Adherent cells were detached using 100 µL of pre-equilibrated trypsin-EDTA solution (0.25% trypsin [wt/vol] and 0.5 mM EDTA in PBS [pH 7.4] lacking metal cations). Following incubation for 5 min at 37°C, the reactions were quenched with 200 µL of 0.85% NaCl, and CFUs were manually enumerated on TSA at 37°C for 18 h.

### *S. aureus* binding to HMEC-1 endothelial cells

The wild-type *S. aureus* JE2 strain and the Δ*fmtA* mutant were grown overnight in TSB at 37°C with aeration at 225 rpm. The overnight cultures were then diluted 1:100 into TSB (5 mM HEPES, pH 7.0) in sterile 13 mL polypropylene tubes with snap caps. The bacteria were then propagated for 2–2.5 h with aeration at 225 rpm at 37ºC. Next, the bacterial cells were harvested, washed once with 1 mL of sterile saline (0.9%, wt/vol NaCl), and resuspended in 1 mL of serum-free RPMI supplemented with 25 mM HEPES. The bacteria were diluted accordingly to generate a bacterial cell suspension yielding an MOI of 10 and 1. Next, 1 mL of the bacterial suspension was applied to 12-well tissue culture plates containing ~4.5 × 10^5^ HMEC-1 cells per well. The tissue culture plates infected with bacteria were incubated for 10 min on an orbital shaker at 50 rpm at 37°C. After 10 min, the plate wells were washed twice with 1 mL of sterile PBS at room temperature. The PBS was aspirated, and the HMEC-1 cells/bacteria were incubated for ~5 min in 0.5 mL of 0.1 (vol/vol) Triton X-100. The lysate was then serially diluted and drop-plated onto TSA to determine the CFU/mL for each lysate. The number of bacteria input for each biological replicate was also determined by serially diluting and drop-plating the starting bacterial suspensions onto TSA. The plates were incubated overnight at 37°C, and the colony-forming units were manually counted. The fraction of bacteria bound was determined by dividing the resulting CFU/mL from each HMEC-1/bacterial lysate by the CFU/mL for each input.

### Measuring the abundance of FnbpA/B on the surface of *S. aureus* cells

FnbpA/B surface abundance was measured using an ELISA as described previously (70), with minor modifications. An MRSA USA300 JE2 strain devoid of SpA and Sbi (JE2-∆*spa* ∆*sbi*) was used to avoid non-specific immunoglobulin binding. Overnight cultures were prepared in biological triplicate for each strain. A single colony was used to inoculate 3 mL of TSB, followed by incubation at 37°C with aeration (220 rpm). Saturated overnight cultures were diluted 1:100 in TSB and incubated at 37°C with shaking (220 rpm) to an OD_600nm_ of 0.4 for optimal FnbpA/B expression (70). The cells were then harvested by centrifugation at 4,000 × *g* at 4°C for 10 min, washed with PBS (pH 7.4), standardized to an OD_600nm_ of 2.0, and 200 µL of the standardized cultures were applied to NuncMaxiSorp microtiter plates (Thermo Scientific). The cells were serially diluted in the microtiter plate using PBS (pH 7.4), followed by incubation for 5 min with vigorous agitation (900 rpm) and then for 1 h with gentle agitation (100 rpm) at room temperature. For the final 20 min of incubation, plate lids were removed, and the cultures were irradiated under ultraviolet light. The plates were then washed with PBS (pH 7.4), as described previously, and 100 µL of PBS (pH 7.4) was applied before the cultures were irradiated again under UV light for 20 min. Following UV treatment, each well was blocked with 300 µL of 2% (wt/vol) BSA in PBS (pH 7.4) for 1 h at room temperature. To ensure the strains adhered equally to the microtiter plates, two identical plates were prepared for all strains: one plate was used to detect CWA protein surface abundance, and the other was used to assess the adherence of the strains using the crystal violet method described above.

To measure the abundance of surface-exposed Fnbps, 100 µL of a mouse anti-FnBPA/B antibody (70) (1:10,000 dilution in 1% [wt/vol] BSA in PBS [7.4]) was applied to the immobilized cells. The plates were then incubated for 5 min with vigorous agitation (600 rpm) and 55 min with gentle agitation (100 rpm) at room temperature, followed by washing with PBS. Next, 100 µL of peroxidase AffiniPure F(ab′)_2_ fragment goat anti-mouse IgG (H + L) HRP-conjugated secondary antibody (1:10,000 in 1% [wt/vol] BSA in PBS [7.4]; Jackson ImmunoResearch) was applied to each well and incubated as described. Following thorough washing of the wells with PBS (pH 7.4), 100 µL of room temperature equilibrated 1-step Ultra TMB (3,3′,5,5′-tetramethylbenzidine) colorimetric substrate (Thermo Scientific) was applied and incubated for 20 min at room temperature. Reaction quenching was achieved by adding 100 µL of 2 M H_2_SO_4_. The Abs_450nm_ was measured using a BioTek Synergy H1 microplate reader.

### Scanning electron microscopy

SEM was carried out as previously described (70). Saturated overnight cultures were diluted 1:100 in TSB and incubated at 37°C with aeration (220 rpm) until an OD_600nm_ of 1.0 was achieved. The cells were harvested by centrifugation, washed three times with sterile 0.85% (wt/vol) NaCl, resuspended in 400 µL of 0.85% (wt/vol) NaCl, and 200 µL of the cell suspension was applied to a carbon planchette (Ted Pella), followed by incubation for 30 min at room temperature. Adhered cells were fixed with 2% glutaraldehyde (vol/vol) for 30 min. The planchettes were washed three times with 800 µL of 0.85% (wt/vol) NaCl and were then submerged in 1% osmium tetroxide for 30 min. After washing once with 800 µL of 0.85% (wt/vol) NaCl, a dehydration series was performed with ethanol, and the planchettes were then fully dried using a Denton DCP-1 critical point dryer. The samples were sputter-coated (Denton Desk V TSC) immediately with 15 nm of gold. Image acquisition was performed at the Advanced Analysis Centre, University of Guelph, using an FEI Quanta FEG 250 scanning electron microscope (Thermo Fisher Scientific) operated at 5.0 kV under high vacuum.

### Citrate-induced biofilm formation

Biofilm assays were conducted as previously described (73) with minor modifications. Briefly, saturated overnight cultures were prepared in TSB for each strain in biological triplicate. The overnight cultures were diluted to an OD_600nm_ of 0.1 in TSB containing 0.2% glucose (wt/vol) and 0.2% sodium citrate (wt/vol). A volume of 100 µL of the diluted cultures was applied to the microtiter plate wells (Costar 3595; Corning Inc.), followed by incubation at 37°C for 6 h in a humidified plastic container. Nonadherent cells were aspirated, and the biofilms were washed with 300 µL of PBS (pH 7.4). The biofilms were dried for 1 h before staining with 0.1% crystal violet solubilized in 7% acetic acid. The Abs_595nm_ was measured using a BioTek Synergy H1 microplate reader. Biofilm experiments were repeated on three separate days, with each experiment consisting of three biological replicates and 18 technical replicates.

## Data Availability

All study data are included in the article and/or SI Appendix.

## SUPPLEMENTAL MATERIAL

Supplemental material mbio.02337-25-s0001.pdf Supplemental figures and tables.

ASM does not own the copyrights to Supplemental Material that may be linked to, or accessed through, an article. The authors have granted ASM a non-exclusive, world-wide license to publish the Supplemental Material files. Please contact the corresponding author directly for reuse.
