# Erratum for Berry et al., “The *Staphylococcus aureus* esterase FmtA is essential for wall teichoic acid D-alanylation”

**DOI:** 10.1128/mbio.01519-26

**Published:** 2026-07-10

**Authors:** Kirsten A. Berry, Mackenzie T. A. Verhoef, Zhiyong Zheng, Ronald S. Flannagan, Telmo O. Paiva, Stephanie E. Gilbert, M. Sameer Al-Abdul-Wahid, David E. Heinrichs, Yves F. Dufrêne, Georgina Cox

## ERRATUM

Volume 16, no. 10, e02337-25, 2025, https://doi.org/10.1128/mbio.02337-25. Introduction, paragraph 4, sentence 3: “the formyl methionyl transferase A (FmtA) enzyme in *S. aureus* has...” should read “the FmtA enzyme, named for its role as a factor that affects the methicillin resistance level and autolysis in the presence of Triton X-100 in *S. aureus*, has….”

