# Supplementary material for: The Staphylococcus aureus esterase FmtA is essential for wall teichoic acid D-alanylation
Source: mBio. 2025 Sep 12;16(10):e02337-25. doi: 10.1128/mbio.02337-25 (PMC12505958; doi:10.1128/mbio.02337-25)
Supplement: Supplemental material — Supplemental figures and tables. [file mbio.02337-25-s0001.pdf]

Supplementary information: The *Staphylococcus aureus* esterase FmtA is essential for wall teichoic acid D-alanylation

Kirsten A. Berry[1*], Mackenzie T.A Verhoef[1*], Zhiyong Zheng[2], Ronald S. Flannagan[3], Telmo O. Paiva[2], Stephanie E. Gilbert[1], M. Sameer Al-Abdul-Wahid[4], David E. Heinrichs[3], Yves F. Dufrêne[2] & Georgina Cox[1]

From the [1]Department of Molecular and Cellular Biology, University of Guelph, 50 Stone Rd E, Guelph, Ontario, Canada N1G 2W1

[2]Louvain institute of Biomolecular Science and Technology, UCLouvain, Croix du Sud, 4-5, bte L7.07.07, B-1348 Louvain-la-Neuve, Belgium

[3]Department of Microbiology and Immunology, University of Western Ontario, London, Ontario, Canada N6A 5C1

[4]Advanced Analysis Centre, University of Guelph, 50 Stone Rd E, Guelph, Ontario, Canada N1G 2W1

[*]These authors contributed equally

Corresponding author: Dr. Georgina Cox, College of Biological Sciences, Department of Molecular and Cellular Biology, University of Guelph, 50 Stone Rd E, Guelph, Ontario, Canada N1G 2W1. Tel: +1 519-824-4120, Email: gcox@uoguelph.ca

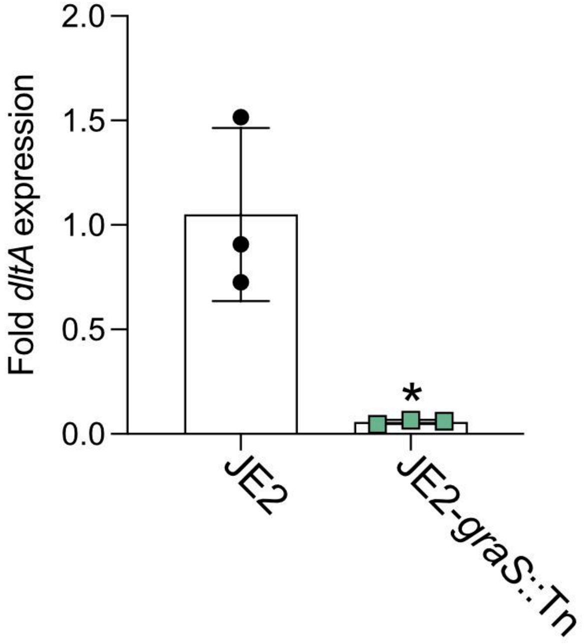

Fig. S1. RT-qPCR analysis revealed decreased *dltA* expression in an MRSA USA300 JE2 clone LAC (JE2) strain with a transposon insertion in *graS* (*graS*::Tn). Relative quantification was calculated using the ΔΔCt Method (Livak Method). Each data point represents a single biological replicate determined by averaging three technical replicates. Error bars represent the standard deviation of the mean. *P*-values were calculated using the two-tailed unpaired Student's *t*-test and were denoted as $P \leq 0.05$*.

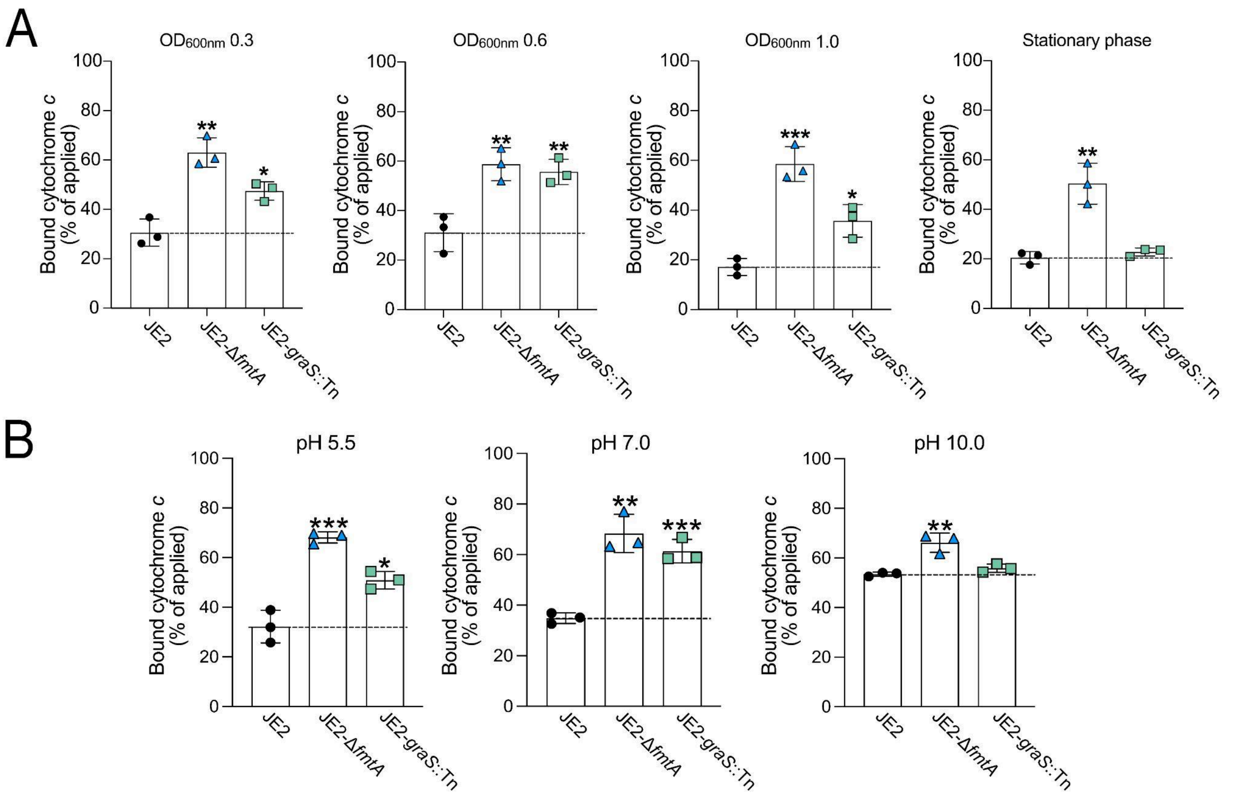

Fig. S2. Relative net cell surface charge of *S. aureus* mutants measured by cytochrome *c* binding across growth phases and pH values. (A) Measuring binding of cytochrome *c* (percent of applied) to MRSA USA300 JE2 clone LAC (JE2) strains sampled at an OD_{600nm} of 0.3, 0.6, 1.0, and the stationary growth phase. **(B)** Measuring binding of cytochrome *c* (percent of applied) to JE2 cells suspended in 100 mM MES [pH 5.5], 5 mM HEPES [pH 7.0], or 50 mM BTP [pH 10.0], respectively, and related to Fig. 1E. Each data point represents a single biological replicate determined by averaging three technical replicates. Error bars represent the standard deviation of the mean. *P*-values were calculated using the two-tailed unpaired Student's *t*-test comparing each mutant to the wild-type strain (JE2) and were denoted as $P \leq 0.05$ *, $P \leq 0.01$**, $P \leq 0.001$***, $P \leq 0.0001$****.

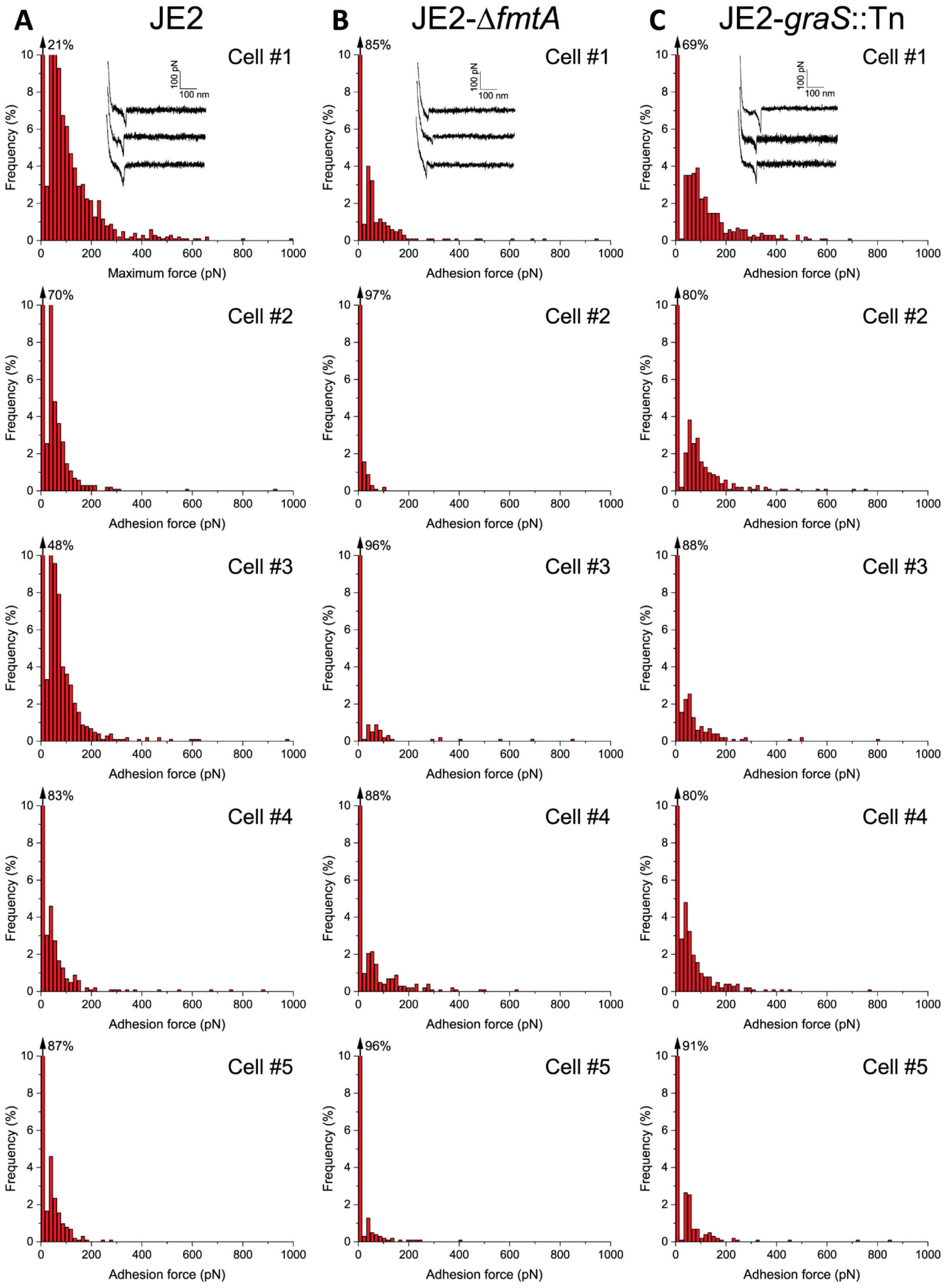

Fig. S3. Adhesion force data measured by recording force-distance curves between negatively charged AFM tips and single bacterial cells. Five representative cells are shown for each strain. Inset: representative adhesion force profiles. Related to Fig. 1.

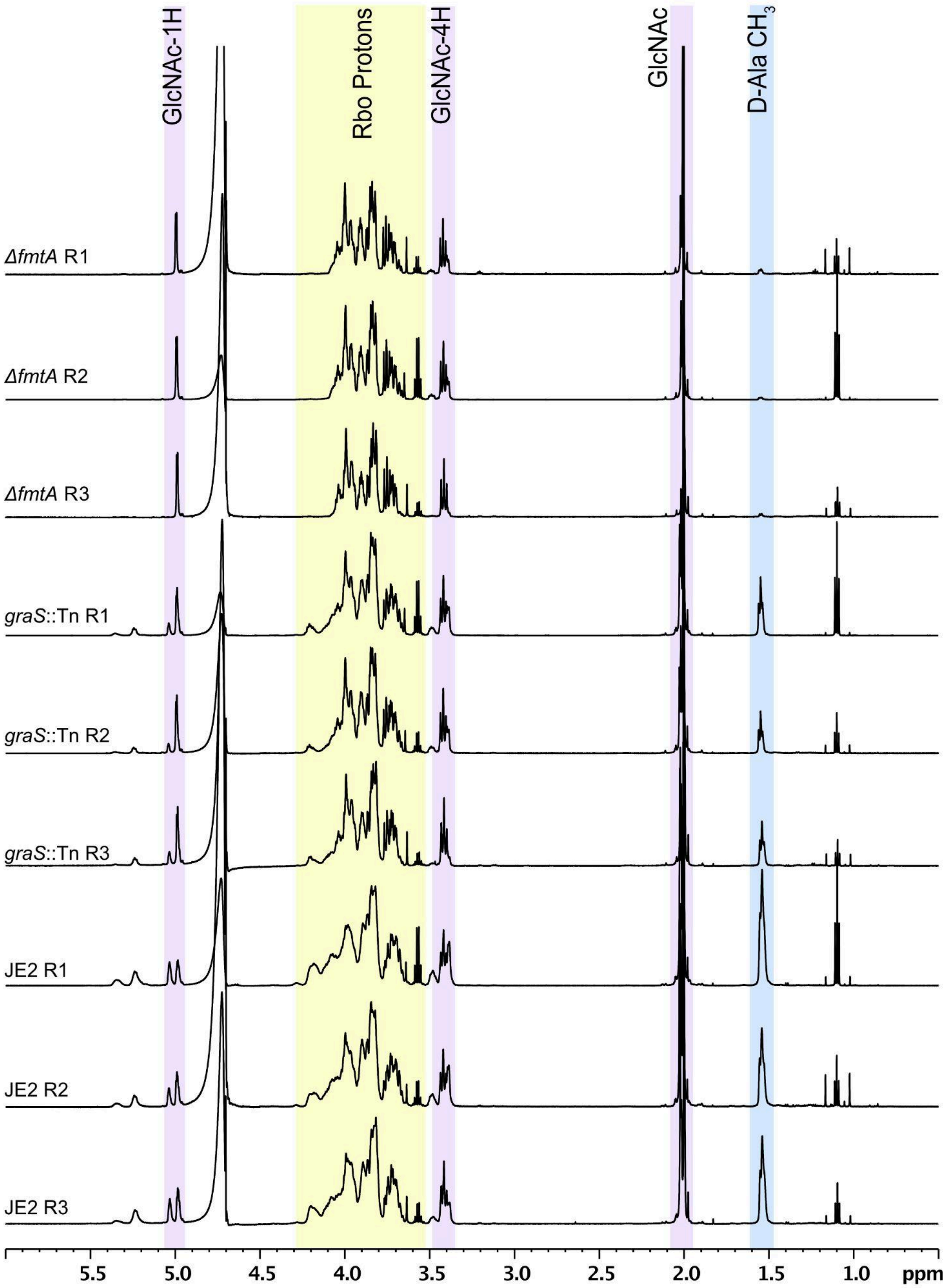

Fig. S4. ^{1}H NMR analysis of *S. aureus* wall acid (WTA) D-alanylation. ^{1}H NMR spectra of WTA extracted from MRSA USA300 JE2 clone LAC (JE2), JE2-*graS*::Tn, and JE2-Δ*fmtA* strains in biological triplicate. Biological replicates (R1-3) were prepared from three independent purifications. All spectra were collected before the addition of NaOH. Related to Fig. 2 and Table 1.

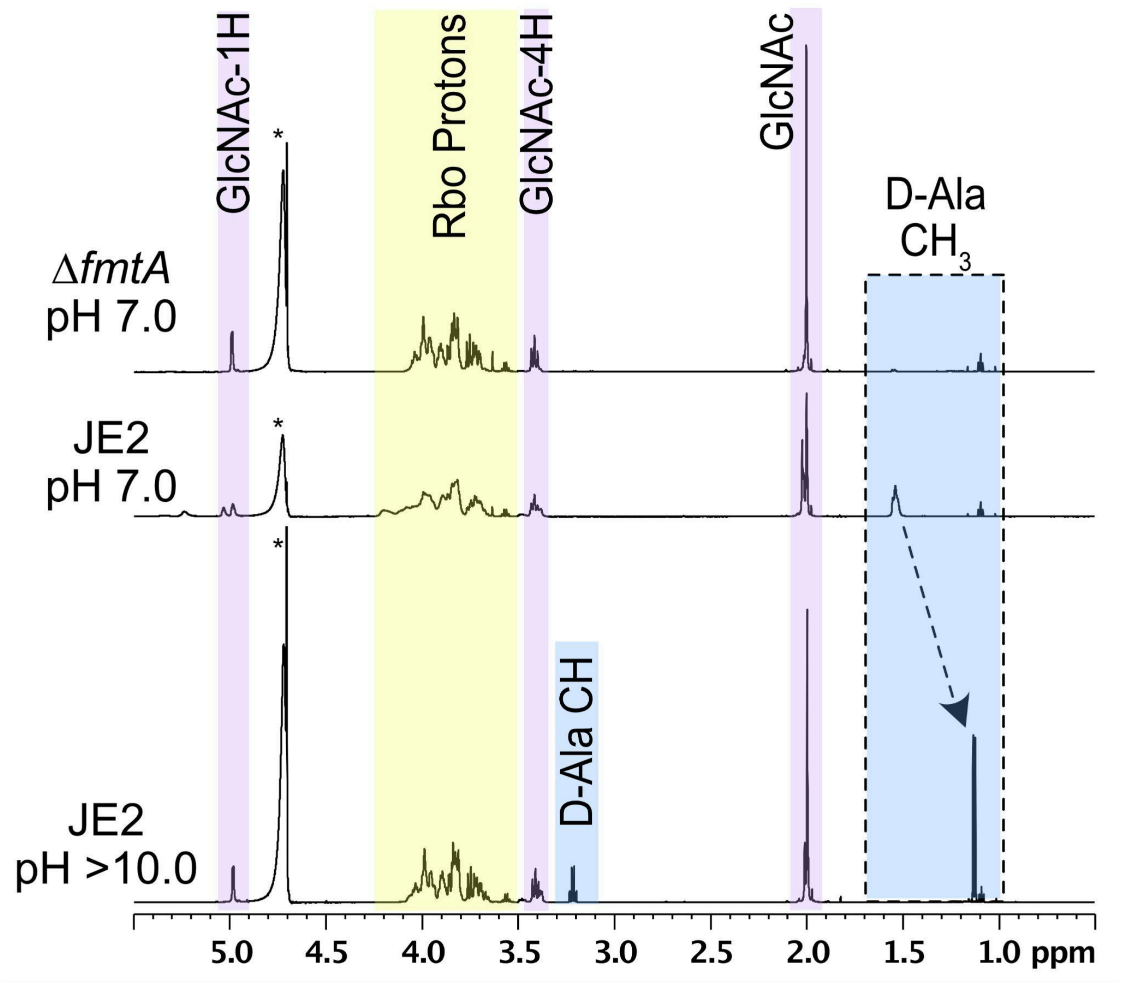

Fig. S5. NaOH induces D-Ala to dissociate from the WTA backbone. ^{1}H NMR spectra of WTA purified from JE2 and Δ*fmtA* before the samples were adjusted to pH 10.0 through the addition of NaOH. The artifact at 4.7 ppm is attributed to incomplete water suppression and is marked with an asterisk. The blue box indicates the region containing the methyl peak of D-Ala. The D-Ala methyl peak shifts from 1.60-1.42 ppm to 1.20-1.10 ppm upon the addition of NaOH (as denoted by the arrow), indicating hydrolysis from the WTA backbone. Related to Fig. S6 and S7.

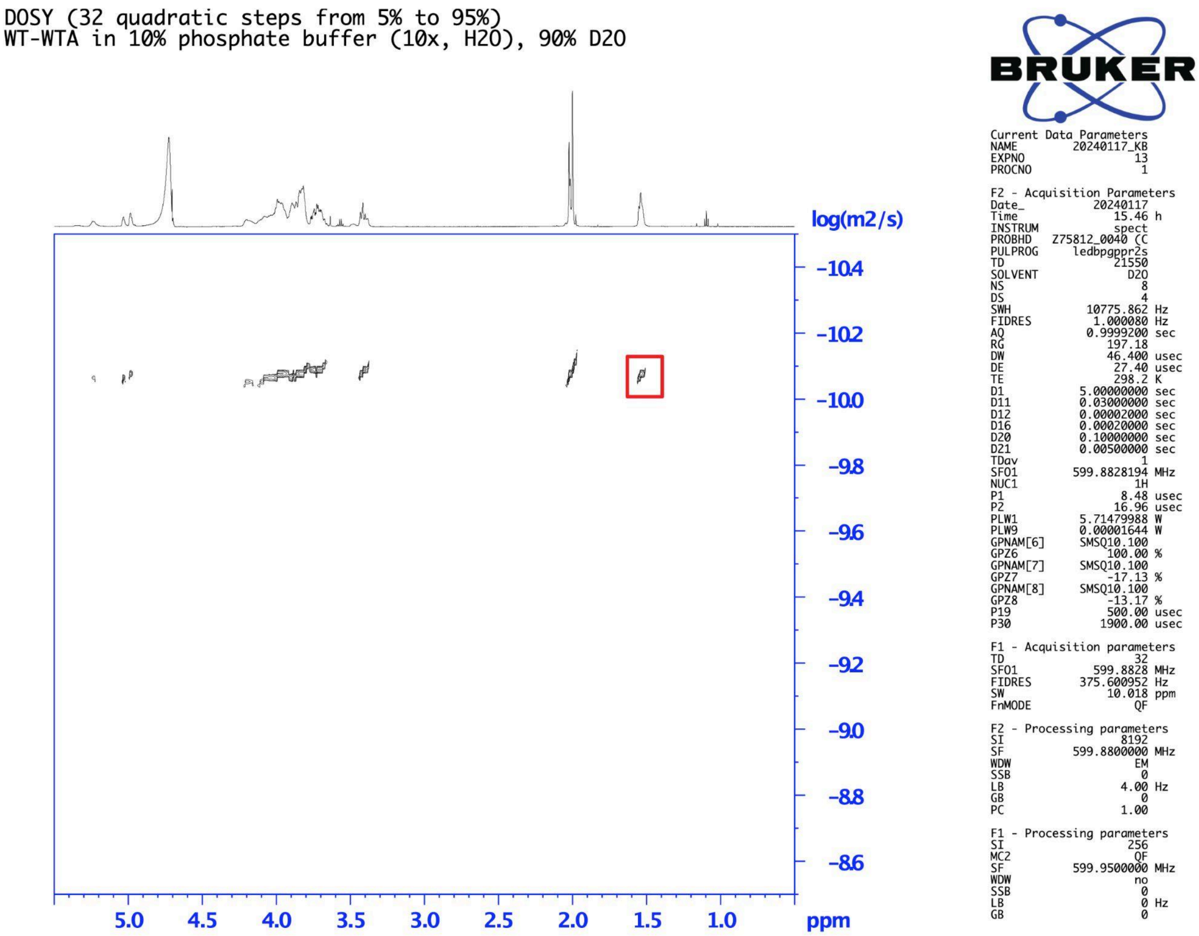

Fig. S6. DOSY spectrum of WTA extracted from MRSA USA300 JE2 clone LAC (JE2) before the sample was adjusted to pH 10.0 through the addition of NaOH. The red box highlights the DOSY peak corresponding to the CH_3 group of D-Ala, which has the same translation diffusion coefficient (y-axis coordinate) as the other WTA peaks in the spectrum, indicating that D-Ala is covalently bound to WTA. The peak corresponding to the CH group of D-Ala overlaps with WTA peaks from the Rbo protons. The corresponding ^1H-^1D NMR spectrum is overlaid on the top axis and is related to Fig. S5.

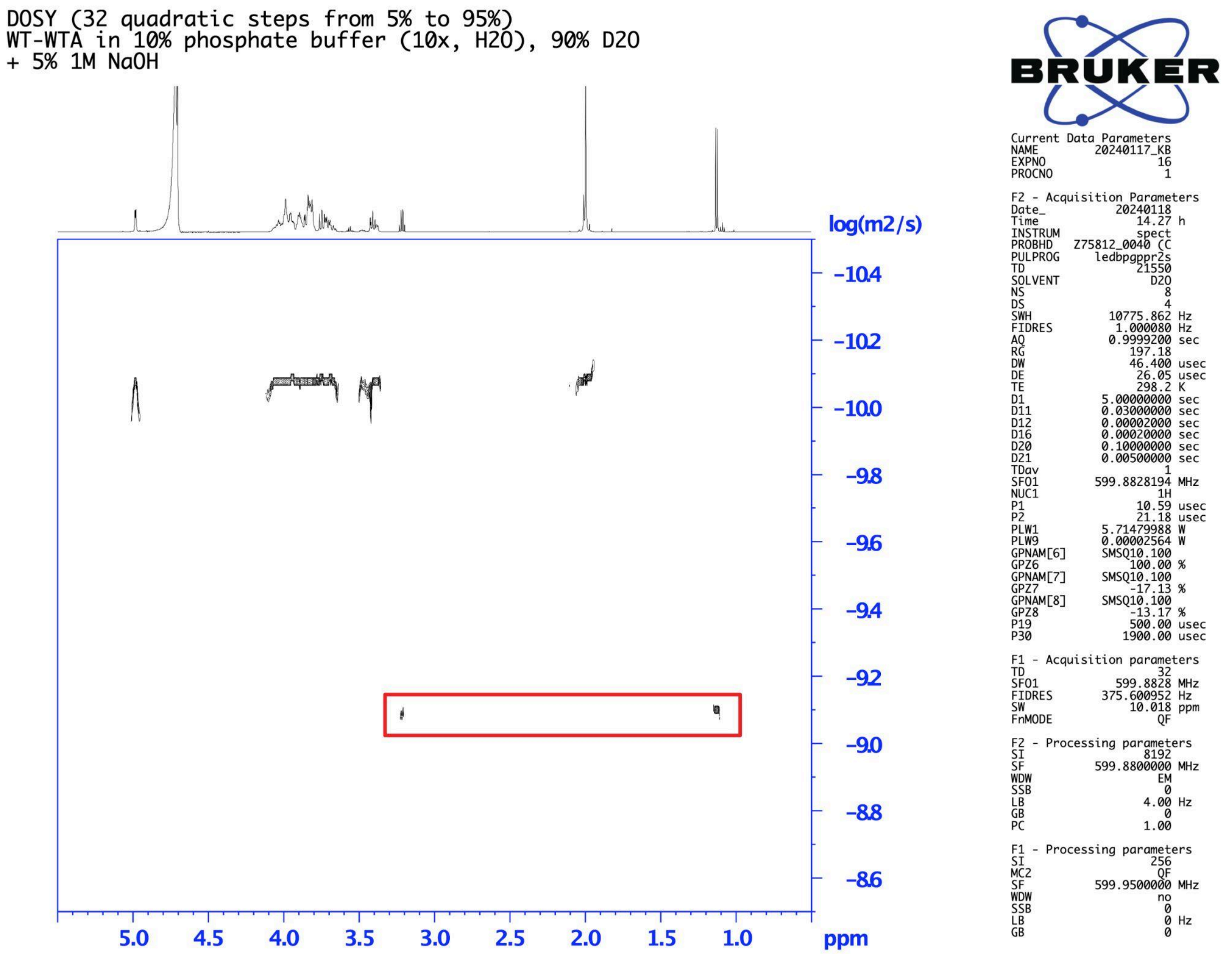

Fig. S7. DOSY spectrum of WTA extracted from MRSA USA300 JE2 clone LAC (JE2) after the sample was adjusted to pH 10.0 through the addition of NaOH. The red box highlights the DOSY peak corresponding to the CH and CH_3 groups of D-Ala, which has a much faster translational diffusion coefficient (lower y-axis coordinate) than the other WTA peaks in the spectrum, confirming that D-Ala is not bound to WTA. Related to Fig. S5.

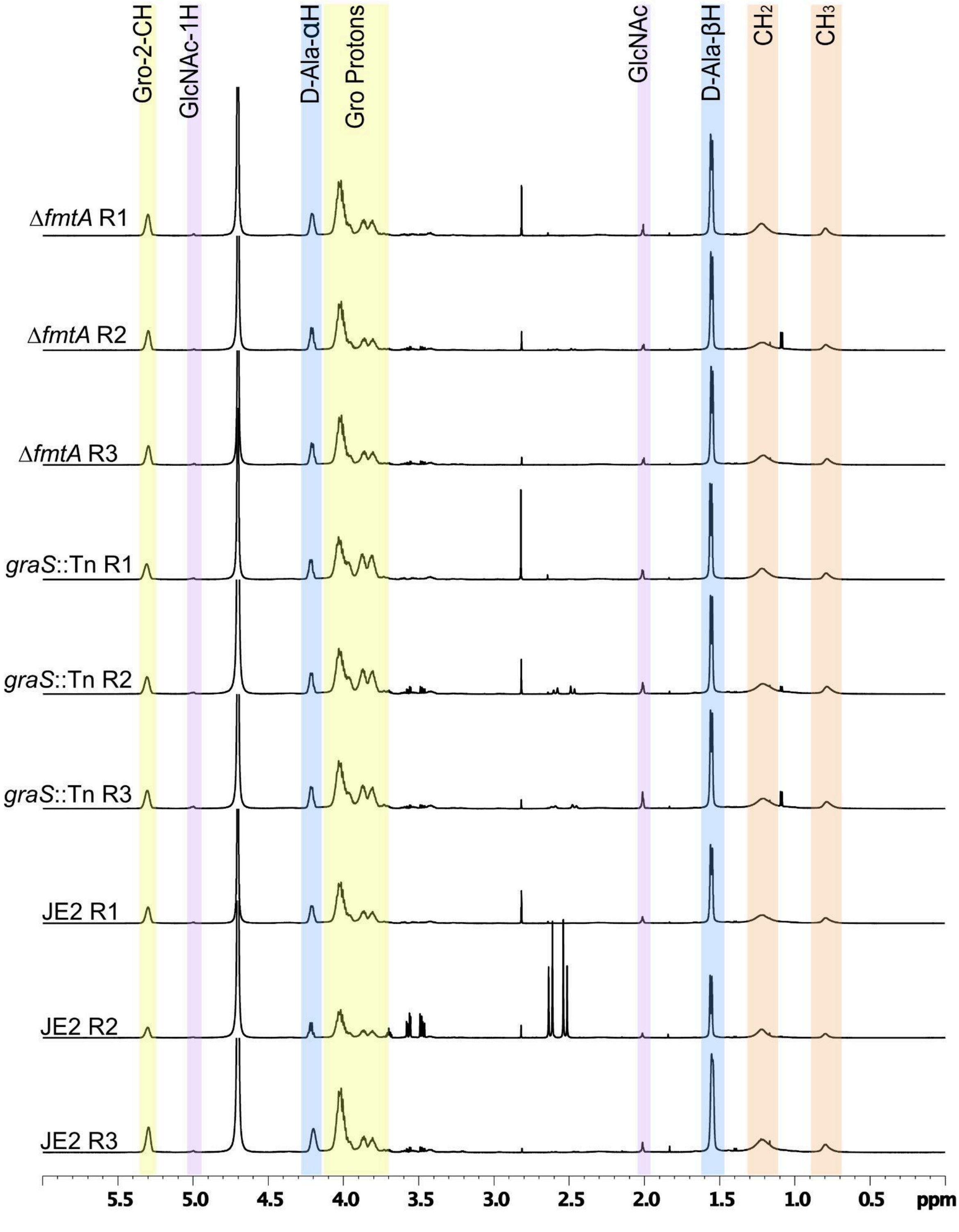

Fig. S8. ^{1}H NMR analysis of lipoteichoic acid (LTA) D-alanylation. ^{1}H NMR spectra of LTA extracted and purified from MRSA USA300 JE2 clone LAC (JE2), JE2-*graS*::Tn, and JE2-Δ*fmtA* in biological triplicate. All samples were prepared on independent days, and all spectra were collected before the addition of NaOH. Related to Fig. 2C.

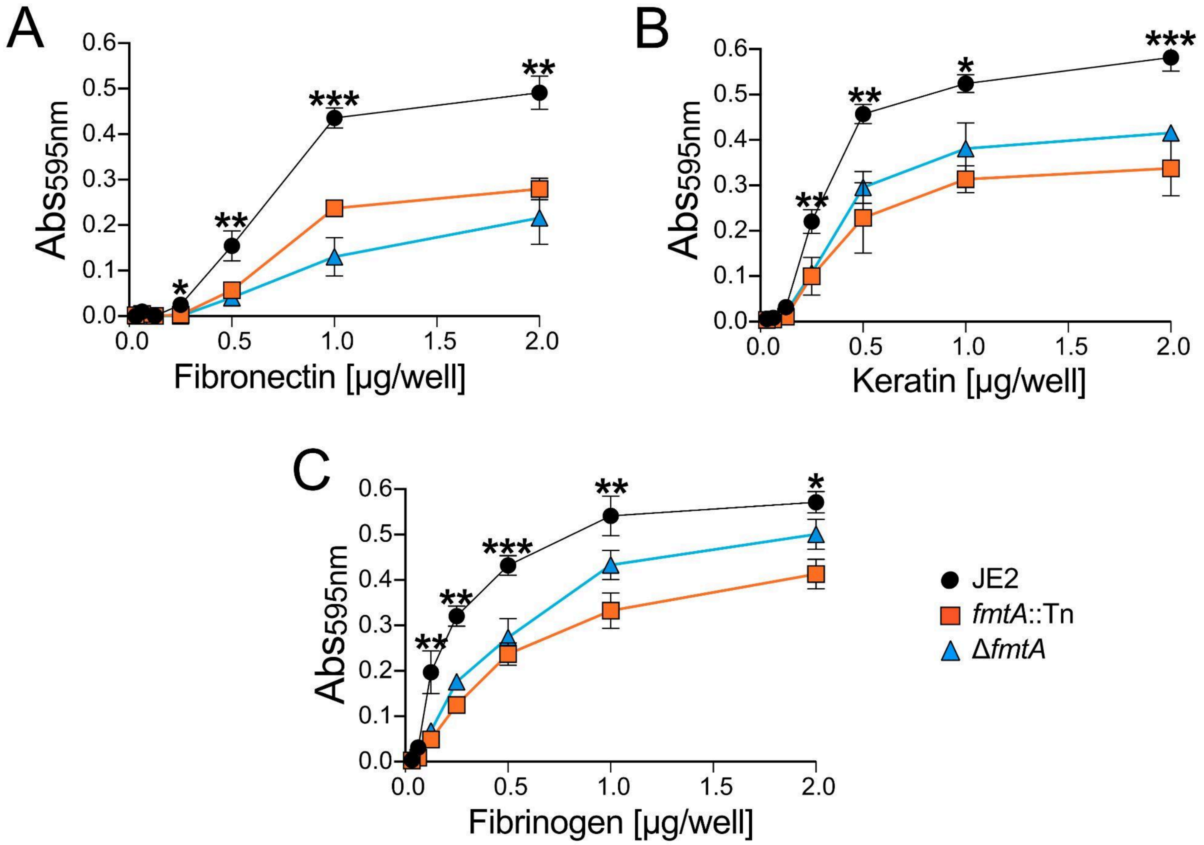

Fig. S9. Disruption of *fmtA* impairs *S. aureus* host ligand adhesion. MRSA USA300 JE2 clone LAC (JE2) (black), JE2-*fmtA*::Tn (orange), and JE2-Δ*fmtA* (blue) adhesion to **(A)** fibronectin, **(B)** keratin, and **(C)** fibrinogen assessed using crystal violet staining (Abs_{595nm}) of adhered cells. Each data point represents the average of three biological replicates, presented as mean ± standard deviation. *P*-values were calculated using the two-tailed unpaired Student's *t*-test comparing the *fmtA* mutant (blue) to the parental strain (JE2). *P*-values are denoted as $P \leq 0.05$ *, $P \leq 0.01$**, $P \leq 0.001$***. Related to Fig. 4.

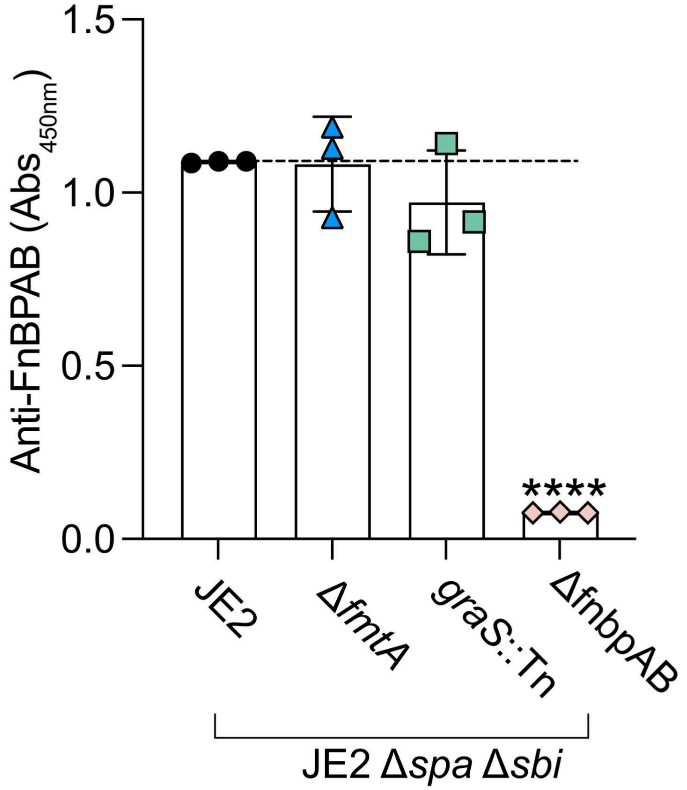

Fig. S10. Disruption of *fmtA* and *graS* does not impact the surface display of the fibronectin-binding proteins A and B (FnbpA/B). ELISA-based (Abs_{450nm}) detection of FnbpA/B on the surface of MRSA USA300 JE2 clone LAC (JE2), JE2-Δ*fmtA,* and JE2-*graS*::Tn. A *fnbpA/B* deficient mutant was used as a negative control. Each data point represents a single biological replicate. Error bars represent the standard deviation of the mean. *P*-values were calculated using the two-tailed unpaired Student's *t*-test comparing each mutant to the parental strain (JE2) and were denoted as $P \leq 0.0001$****.

Table S1. Strains and plasmids used in this study		
**Strain/Plasmid**	**Description**	**Source**
**Strains**		
S. aureus		
**USA300 JE2 (JE2)**	Wildtype USA300 strain LAC cured of resistance plasmids, CA-MRSA	BEI Resources; (1)
**JE2-Δ*fmtA***	USA300 JE2 *fmtA* deletion	This study
**JE2-Δ*fmtA* Δ*spa* Δ*sbi***	USA300 JE2 *fmtA, spa,* and *sbi* deletions	This study
**JE2-*fmtA*::Tn**	USA300 JE2 with a transposon insertion in the *fmtA* gene, Ery^r	BEI Resources; (1, 2)
**JE2-*graS*::Tn**	USA300 JE2 with a transposon insertion in the *graS* gene, Ery^r	BEI Resources; (1)
**Δ*graS***	USA300 with Δ*graS* created by pKOR mutagenesis	(3)
**JE2-*graS*::Tn Δ*spa* Δ*sbi***	USA300 JE2-*graS*::Tn devoid of *spa* and *sbi* genes, Ery^r	This study
**JE2-Δ*spa* Δ*sbi***	USA300 JE2 *spa* and *sbi* deleted	(4)
**JE2-Δ*fnbAB* Δ*spa* Δ*sbi***	USA300 JE2 *fnbpA, fnbpB, sbi* and *spa* deleted	(5)
**RN4220**	Inactivated restriction-modification system, capable of accepting foreign DNA	(6, 7)
**RN4220-*fmtA*::Tn**	RN4220 with a transposon insertion in *fmtA,* Ery^r	This study
**USA300 AH1263**	Wildtype USA300 LAC-derived strain	(8)
**AH1263-*fmtA*::Tn**	USA300 AH1263 with a transposon insertion in *fmtA,* Ery^r	This study
E. coli		
**DH5α**	*endA*$^-$ routine cloning host for high-quality DNA preparations	ThermoFisher Scientific
**IM08B**	*S. aureus* methylation machinery, SA08BWPN25-hsdS (CC8-1) (SAUSA300_0406) of NRS384 integrated between *essQ* and *cspB*	BEI Resources; (9)

**Plasmids**		
**pJB38**	Amp^r (*E. coli*), Cam^r (*S. aureus*), pCL10 with Pxyl/tetO-secY570 and *Xho*I site removed, temperature-sensitive	BEI Resources; (10)
**pJB38:*fmtA* AD**	Amp^r (*E. coli*), Cam^r (*S. aureus*), temperature-sensitive pJB38 modified by insertion of the *fmtA* fusion	This study
**pJB38:*spa* AD**	Amp^r (*E. coli*), Cam^r (*S. aureus*), temperature-sensitive pJB38 modified by insertion of the *spa* fusion	(5)
**pJB38:*sbi* AD**	Amp^r (*E. coli*), Cam^r (*S. aureus*), temperature-sensitive pJB38 modified by insertion of the *sbi* fusion	(5)
**pALC2073**	Amp^r (*E. coli*), Cam^r (*S. aureus*), multicopy shuttle vector with a TetR-inducible Pxyl/tetO promoter	(11)
**pALC2073:*fmtA***	Amp^r (*E. coli*), Cam^r (*S. aureus*), pALC2073 expressing *fmtA* from USA300 JE2	This study
**pALC2073:*graS***	Amp^r (*E. coli*), Cam^r (*S. aureus*), pALC2073 expressing *graS* from USA300 JE2	(3)

Ery^r, erythromycin resistance; Cam^r, chloramphenicol resistance; Amp^r, ampicillin resistance

Table S2. Primers used in this study

Primers/ Oligonucleotides	Sequence (5' - 3')	Source
Upstream	CTCGATTCTATTAACAAGGG	(12)
Buster	GCTTTTTCTAAATGTTTTTTAAGTAAATCAAGTAC	(12)
GraS_Tn_Check	CACTGCATTATGGGATGATG	This study
PrimerA_FmtA	TCAGGAGCTCTTGTTCTTCGGGATTGAC	This study
PrimerB_FmtA	GTCAGATAAGGTCGATGCGCCTCACAATGTATTCAT	This study
PrimerC_FmtA	ATGAATACATTGTGAGGCGCATCGACCTTATCTGAC	This study
PrimerD_FmtA	TCAGGTCGACGGACGTTTACAAACATTC	This study
PrimerA_Spa	TCAGGAGCTCACGGTGGTTTGACTGTAG	(5)
PrimerB_Spa	CATACAGGGGGTATTAATAAACAAACAATACACAACG	(5)
PrimerC_Spa	CGTTGTGTATTGTTTGTTTATTAATACCCCCTGTATG	(5)
PrimerD_Spa	TCAGGTCGACGTAGAATTCACAATTCTAGC	(5)
PrimerA_Sbi	TCAGGAGCTCAGACATGCGTTGAACCAC	(5)
PrimerB_Sbi	GAGAAGATATTTTTGATTGAGTGTATTCCCTTTCTTTTTAC	(5)
PrimerC_Sbi	GTAAAAAGAAAGGGAATACACTCAATCAAAAATATCTTCT C	(5)
PrimerD_Sbi	TCAGGTCGACTAATCGTTTGCTAGTAATG	(5)
pJB38_Fwd_NEW	GGGTTCCGCGCACATTTC	(5)
pJB38_Rvs_NEW	TAAGGGTAACTAGCCTCGC	(5)
FmtA_Fwd_Comp	TTTTTTGAGCTCCGAGTGATTTATACAAAGTGATAAGG	This study
FmtA_Rvs_Comp	TTTTTTGAATTCGCCTTAATGCCATATACATG	This study
FmtA_Fwd_Check	GTGGATCGTAATAAGGAAAAG	This study
FmtA_Rvs_Check	CAATGGGATAGGCTTAGATG	This study
DltA_Fwd_qPCR	CGCCAGTCTGAGTTCGTAAA	This study
DltA_Rvs_qPCR	GTCATTTCTGCATTGTCCGTAAC	This study
RpoD(SigA)_Fwd_qPCR	GACCCAGTTCGTATGTACCTTAAA	This study
RpoD(SigA)_Rvs_qPCR	GCTACTTCATCACCTTGTTCAATAC	This study

Supplemental References:

1. P. D. Fey, *et al.*, A genetic resource for rapid and comprehensive phenotype screening of nonessential *Staphylococcus aureus* genes. *mBio* (2013). Available at: http://dx.doi.org/10.1128/mbio.00537-12.

2. L. E. Petrie, A. C. Leonard, J. Murphy, G. Cox, Development and validation of a high-throughput whole-cell assay to investigate *Staphylococcus aureus* adhesion to host ligands. *J. Biol. Chem.* (2020). https://doi.org/10.1074/jbc.RA120.015360.

3. R. S. Flannagan, R. C. Kuiack, M. J. McGavin, D. E. Heinrichs, *Staphylococcus aureus* uses the GraXRS regulatory system to sense and adapt to the acidified phagolysosome in macrophages. *MBio* **9** (2018).

4. M. I. Goncheva, *et al.*, Stress-induced inactivation of the *Staphylococcus aureus* purine biosynthesis repressor leads to hypervirulence. *Nat. Commun.* **10**, 775 (2019).

5. A. C. Leonard, *et al.*, Autolysin-mediated peptidoglycan hydrolysis is required for the surface display of *Staphylococcus aureus* cell wall-anchored proteins. *Proc. Natl. Acad. Sci. U. S. A.* **120**, e2301414120 (2023).

6. D. Nair, *et al.*, Whole-genome sequencing of *Staphylococcus aureus* strain RN4220, a key laboratory strain used in virulence research, identifies mutations that affect not only virulence factors but also the fitness of the strain. *J. Bacteriol.* **193**, 2332–2335 (2011).

7. I. R. Monk, T. J. Foster, Genetic manipulation of Staphylococci-breaking through the barrier. *Front. Cell. Infect. Microbiol.* **2**, 49 (2012).

8. Z. R. DeMars, *et al.*, Fatty acids can inhibit *Staphylococcus aureus* SaeS activity at the membrane independent of alterations in respiration. *Mol. Microbiol.* **116**, 1378–1391 (2021).

9. I. R. Monk, J. J. Tree, B. P. Howden, T. P. Stinear, T. J. Foster, Complete bypass of restriction systems for major *Staphylococcus aureus* lineages. mBio. 2015; 6 (3): e00308–15. *Epub 2015/05/28. https://doi. org.*

10. J. L. Bose, P. D. Fey, K. W. Bayles, Genetic tools to enhance the study of gene function and regulation in *Staphylococcus aureus*. *Appl. Environ. Microbiol.* **79**, 2218–2224 (2013).

11. R. M. Corrigan, T. J. Foster, An improved tetracycline-inducible expression vector for *Staphylococcus aureus*. *Plasmid* **61**, 126–129 (2009).

12. T. Bae, E. M. Glass, O. Schneewind, D. Missiakas, Generating a collection of insertion mutations in the *Staphylococcus aureus* genome using *bursa aurealis*. *Methods Mol. Biol.* **416**, 103–116 (2008).
